# Success of debridement, antibiotics, and implant retention for acute hematogenous periprosthetic joint infection: a systematic review and meta-analysis

**DOI:** 10.5194/jbji-11-673-2026

**Published:** 2026-11-09

**Authors:** Sina Esmaeili, Mohammadreza Razzaghof, Mohammad Ayati Firoozabadi, Seyed Mohammad Javad Mortazavi

**Affiliations:** 1 Joint Reconstruction Research Center, Tehran University of Medical Sciences, Tehran, Iran; 2 Department of Orthopedic Surgery, Imam Khomeini Hospital Complex, Tehran University of Medical Sciences, Tehran, Iran

## Abstract

**Background**: Acute hematogenous periprosthetic joint infection after hip or knee arthroplasty is difficult to treat. The method of debridement, antibiotics, and implant retention (DAIR) is widely used, but reported success rates vary, and predictors of failure remain unclear. **Methods**: We searched PubMed, Scopus, Web of Science, and Embase from inception through 26 November 2025 for studies reporting outcomes of DAIR in acute hematogenous periprosthetic joint infection. Two reviewers independently screened studies, extracted data, and assessed methodological quality using Joanna Briggs Institute tools. The primary outcome was pooled treatment success. **Results**: Forty-nine studies were included in the qualitative synthesis. Reported success rates ranged from 20 % to 100 %, with a pooled success rate of 64.5 % (95 % confidence interval, 59.3 %–69.4 %; $I^{2}=72$ %, $\tau^{2}=0.44$ ) in the meta-analysis. This pooled estimate describes an average across heterogeneous cohorts and should not be used as a patient-specific prediction. Success was higher in total hip arthroplasty than in total knee arthroplasty (69.4 % vs. 63.2 %). Outcomes improved over time, from 44.9 % before 2010 to 68.7 % in 2020–2025. *Staphylococcus aureus* infection and prolonged symptom duration before debridement were the most consistently reported predictors of treatment failure, whereas some included studies reported improved outcomes with modular component exchange. **Conclusion**: DAIR controls infection in about two-thirds of patients with acute hematogenous periprosthetic joint infection. Failure remains common, especially with *S. aureus* infection or delayed intervention, supporting early diagnosis, prompt surgery, and careful patient selection.

## 1 Introduction

Periprosthetic joint infection (PJI) is among the most serious complications after hip and knee arthroplasty, causing substantial morbidity, impaired quality of life, prolonged hospitalization, and major healthcare costs (Premkumar et al., 2021; McMaster Arthroplasty, 2020). As the volume of primary arthroplasty continues to increase, the burden of PJI is expected to rise accordingly. International registry data suggest infection rates of approximately 1 % after total hip and knee arthroplasty, although reported estimates vary depending on diagnostic criteria, registry methodology, and follow-up duration (Marang-van de Mheen et al., 2017). Acute hematogenous PJI, which accounts for approximately 20 % to 35 % of all cases, occurs when bacteria disseminate from a distant infectious focus or, less commonly, from a contiguous site and seed a previously well-functioning joint implant, often leading to abrupt pain, swelling, and functional decline (Urish et al., 2018; Konigsberg et al., 2014).

Several surgical strategies are available for the management of PJI, including debridement with implant retention and one-stage-revision and two-stage-revision arthroplasty (Fink et al., 2017; Osmon et al., 2013b). In acute hematogenous periprosthetic joint infection, the method of debridement, antibiotics, and implant retention (DAIR) is often the preferred initial approach because it can preserve well-fixed components and avoid more extensive reconstruction (Osmon et al., 2013b). However, reported success rates vary widely, definitions are inconsistent, and focused synthesis in this specific setting remains limited.

A clearer estimate of treatment success, along with a better understanding of reported failure predictors, may help guide patient selection, timing of intervention, and multidisciplinary management.

The aim of this systematic review and meta-analysis was to estimate the pooled success rate of DAIR for acute hematogenous PJI after hip or knee arthroplasty and to identify reported predictors of treatment failure.

## 2 Methods

This systematic review was conducted in accordance with the Preferred Reporting Items for Systematic Reviews and Meta-Analyses (PRISMA) guidelines (Page et al., 2021). The review protocol was prospectively registered in PROSPERO (registration ID: CRD420251245222).

### 2.1 Search strategy

We searched PubMed, Scopus, Web of Science, and Embase from database inception through 26 November 2025 using controlled vocabulary and free-text terms for periprosthetic joint infection and hematogenous infection. Reference lists of included studies and relevant reviews were also screened. No language restriction was applied during the search, but non-English full-text articles were excluded during screening. Full search strategies are provided in Table S1 in the Supplement.

### 2.2 Study selection

Eligible studies reported outcomes of debridement, antibiotics, and implant retention in acute hematogenous periprosthetic joint infection after hip or knee arthroplasty. Retrospective and prospective cohort studies, case control studies, cross-sectional studies, case series, and randomized trials were eligible. Studies had to provide separate extractable outcomes for acute hematogenous cases treated with DAIR. Mixed acute/chronic cohorts or mixed treatment cohorts were excluded unless relevant subgroup data were extractable. Review articles, case reports, congress abstracts, letters, and non-English publications were excluded.

Two reviewers independently screened titles, abstracts, and full texts using Covidence. Inter-rater agreement was quantified with Cohen's κ statistic at the title/abstract stage ( κ=0.73 ) and full-text stage ( κ=0.88 ). Disagreements were resolved by discussion, with adjudication by a senior author when required.

### 2.3 Definition of acute hematogenous PJI

Definitions of acute hematogenous PJI varied across studies. Study-specific definitions were accepted when the infection was explicitly described as acute hematogenous or when the clinical scenario was consistent with hematogenous seeding of a previously well-functioning implanted joint. Definitions and time thresholds from index arthroplasty were recorded during extraction, and subgroup analyses were performed by study definition where feasible.

### 2.4 Data extraction

Two reviewers independently extracted study characteristics, patient characteristics, definitions of acute hematogenous infection, microbiological findings, intervention details, and outcomes using a standardized spreadsheet. All reported predictors of failure were extracted; predictors from multivariable analyses were prioritized over those reported only in univariable analyses. Discrepancies were resolved by consensus.

### 2.5 Assessment of study quality

Methodological quality was assessed independently by two reviewers using the 10-item Joanna Briggs Institute (JBI) critical appraisal checklist (Munn et al., 2020). Because most included studies were retrospective case series or cohort studies, the findings of this review were interpreted with caution, and greater emphasis was placed on the consistency of results across studies rather than on any single study estimate.

### 2.6 Data analyses

A meta-analysis of proportions was performed in R (version 4.2.2) using the metaprop function from the meta package with a generalized linear mixed model and logit transformation. Because substantial between-study variability was expected, pooled estimates were interpreted within a random-effects framework. Heterogeneity was assessed with Cochran's Q test and the $I^{2}$ statistic. Subgroup analyses were conducted by joint type, study definition, publication period, and region. A restrictive sensitivity analysis was performed by limiting the analysis to studies that provided detailed diagnostic criteria for acute hematogenous PJI, included at least 10 patients with acute hematogenous PJI, and reported a minimum follow-up of 1 year to allow adequate assessment of treatment failure. Exploratory meta-regression was performed where sufficient data were available to investigate associations between study-level DAIR success rates and prespecified clinical and procedural characteristics, including mean symptom-to-DAIR interval, modular component exchange rate, proportion of *Staphylococcus aureus* infections, joint age, and mean duration of antibiotic therapy. These models were adjusted for joint type. Publication bias was assessed by means of funnel plot inspection and Egger's regression test, interpreted cautiously because of substantial heterogeneity.

## 3 Results

### 3.1 Study selection

After duplicates were removed, 1002 records underwent title and abstract screening. Ninety-four full-text articles were assessed for eligibility, and 49 studies were ultimately included in the final synthesis (Liukkonen et al., 2024; Soundarrajan et al., 2023; Chen et al., 2021; Huotari et al., 2023; Rahardja et al., 2023; Karlsen et al., 2025; Tarity et al., 2021; Geurts et al., 2013; Westberg et al., 2023; Segawa et al., 1999; Bryan et al., 2017; Chiu and Chen, 2007; Cook et al., 2007; Crockarell et al., 1998; Tsukayama et al., 1996; Estes et al., 2010; Fink et al., 2017; Flierl et al., 2017; Hernandez et al., 2019; Uriarte et al., 2019; Engen et al., 2024; Nurmohamed et al., 2021; Kim et al., 2011; Koh et al., 2015; Konigsberg et al., 2014; Koyonos et al., 2011; Son et al., 2017; Kuo et al., 2019; Lora-Tamayo et al., 2013; Salmons et al., 2023; Renz et al., 2022; Lora-Tamayo et al., 2017; Shao et al., 2022; Chaiyakit et al., 2021; Iza et al., 2019; Pérez-Prieto et al., 2023; Chang et al., 2022; Toh et al., 2021; Sendi et al., 2017; Shohat et al., 2019; Swenson et al., 2018; Vilchez et al., 2011; Waldman et al., 2000; Wouthuyzen-Bakker et al., 2019a; Zhu et al., 2021; Chandler et al., 2025; Liukkonen et al., 2025; Sigmund et al., 2024; Terhune et al., 2025). The study selection process is summarized in the PRISMA flow diagram (Fig. 1).

**Figure 1:**
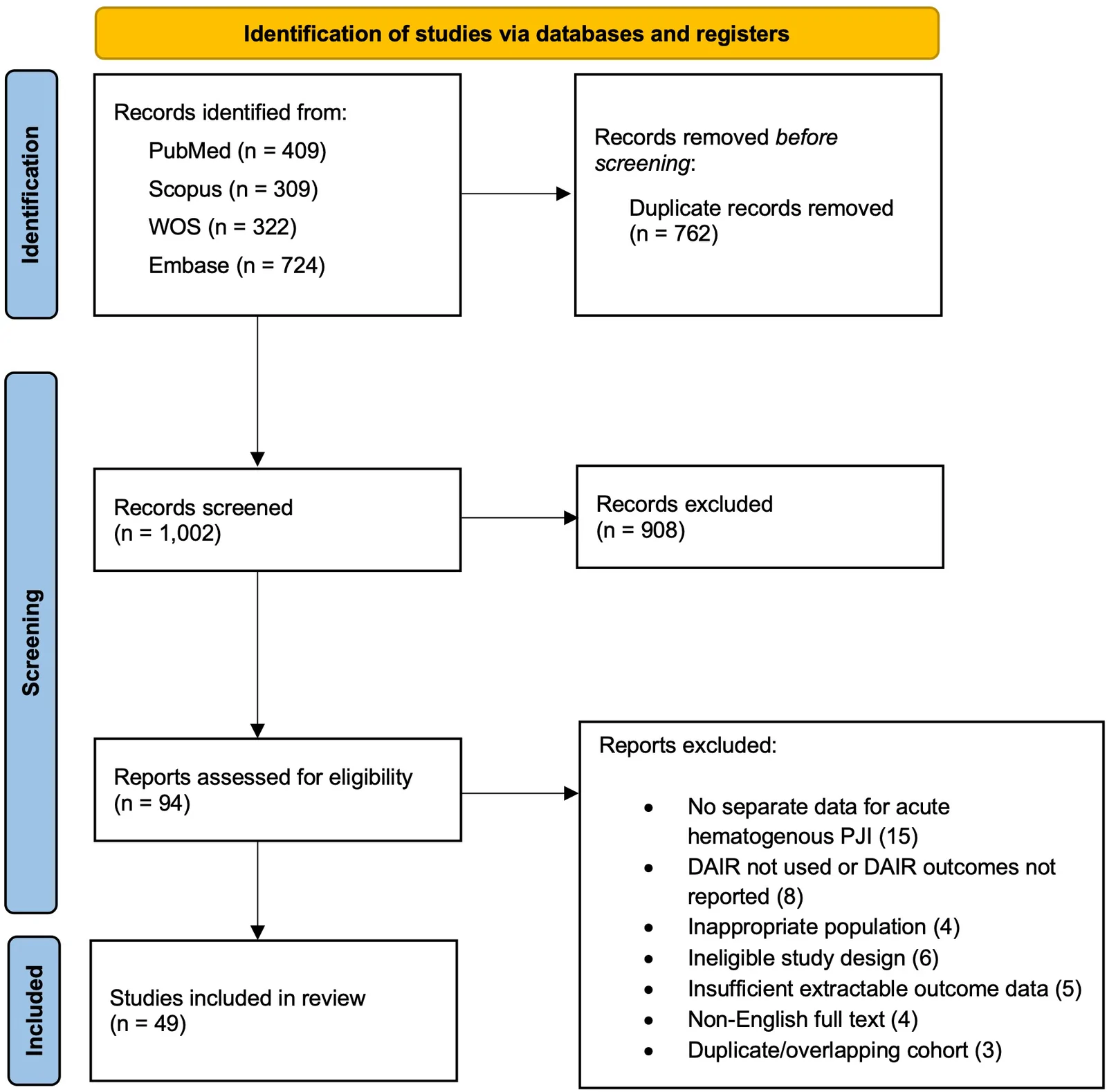
PRISMA flow diagram illustrating the study selection process.

### 3.2 Study characteristics

Forty-nine studies comprising 2318 patients were included in the qualitative synthesis; however, only studies with extractable outcome data were included in each quantitative analysis, resulting in smaller denominators for some pooled estimates.

*Study design and geography.* Among the included studies, 42 were retrospective and 7 were prospective. The studies were conducted across multiple countries, most commonly the United States (19 studies), Spain (6 studies), and Korea (4 studies) (Table 1).

*Patient and joint characteristics.* The review included a total of 2318 patients with acute hematogenous PJI. Among the 21 studies reporting sex distribution, 49 % of patients were men (752 of 1529). Thirty-two studies reported mean age, which ranged from 59 to 74 years, and mean follow-up duration ranged from 0.3 to 8.5 years. Twenty-four studies included both hip and knee arthroplasties, 18 focused exclusively on knee arthroplasty, and 7 focused exclusively on hip arthroplasty (Table 1).

*Definitions of acute hematogenous PJI.* The definition of acute hematogenous PJI varied across studies. Twenty-one studies defined it as infection occurring more than 4 weeks after the index procedure, 18 used a threshold of more than 3 months, and 3 used more than 6 months, whereas 8 studies did not report a specific definition (Table 1). In most studies, symptom duration of fewer than 4 weeks was considered to be consistent with an acute presentation. Thirty-six studies provided detailed diagnostic criteria for acute hematogenous PJI that incorporated features consistent with the concepts described by the International Consensus Meeting (ICM) (Elkins et al., 2019) and the Infectious Diseases Society of America (IDSA) (Osmon et al., 2013a), including a previously well-functioning prosthetic joint and clinical or microbiologic evidence suggestive of hematogenous seeding, such as positive blood cultures or a distant infectious focus.

### 3.3 Quality assessment

Among the included studies, 42 were retrospective, and 7 were prospective. Overall, methodological quality was acceptable according to the JBI critical appraisal tool; however, common limitations included the predominance of retrospective designs, inconsistent reporting of clinical variables, and incomplete reporting across some appraisal domains. Notably, only 8 of 49 studies (16 %) reported clinical information clearly (JBI item 7), representing the main weakness identified in the quality assessment. Table 2 summarizes the JBI appraisal by domain, and study-level ratings are provided in Table S2.

### 3.4 Infective organisms

*S. aureus* was the most frequently reported pathogen, with a pooled prevalence of 34.2 % (95 % CI: 28.5 %–40.3 %; $I^{2}=71$ %, $\tau^{2}=0.47$ ; Fig. S1 in the Supplement). Streptococcal species were the second most common organisms, with a pooled prevalence of 23.6 % (95 % CI: 17.7 %–30.8 %; $I^{2}=70$ %, $\tau^{2}=0.49$ ; Fig. S2). Polymicrobial infections accounted for 8.0 % of reported cases (95 % CI: 4.9 %–12.8 %; $I^{2}=83$ %, $\tau^{2}=0.28$ ; Fig. S3), whereas culture-negative infections represented 17.4 % (95 % CI: 11.1 %–26.2 %; $I^{2}=63$ %, $\tau^{2}=0.57$ ; Fig. S4).

Among studies reporting treatment timing, the interval from symptom onset to DAIR ranged from 3.3 to 15 d, and the time from index arthroplasty to DAIR ranged from 1.3 to 6.5 years. In most studies, modular components were exchanged in all patients undergoing DAIR. Intravenous antibiotic therapy typically lasted 6 weeks, although the duration of subsequent oral antibiotic therapy varied considerably across studies (Table S3).

### 3.5 Treatment success rate

Definitions of treatment success generally required the resolution of clinical symptoms and normalization of laboratory inflammatory markers (e.g., ESR, CRP, synovial fluid analysis), whereas treatment failure consisted of recurrent infection, need for reoperation, or death attributable to infection (Table S4). Across all studies, success rates ranged from 20 % to 100 %, with a pooled success proportion of 64.5 % (95 % CI: 59.3 %–69.4 %; $I^{2}=72$ %, $\tau^{2}=0.44$ ; Fig. 2). This suggests that approximately one in three patients undergoing DAIR for acute hematogenous PJI may ultimately experience treatment failure. Egger's regression test revealed no significant publication bias ( P=0.13 ) (Fig. S5).

**Figure 2:**
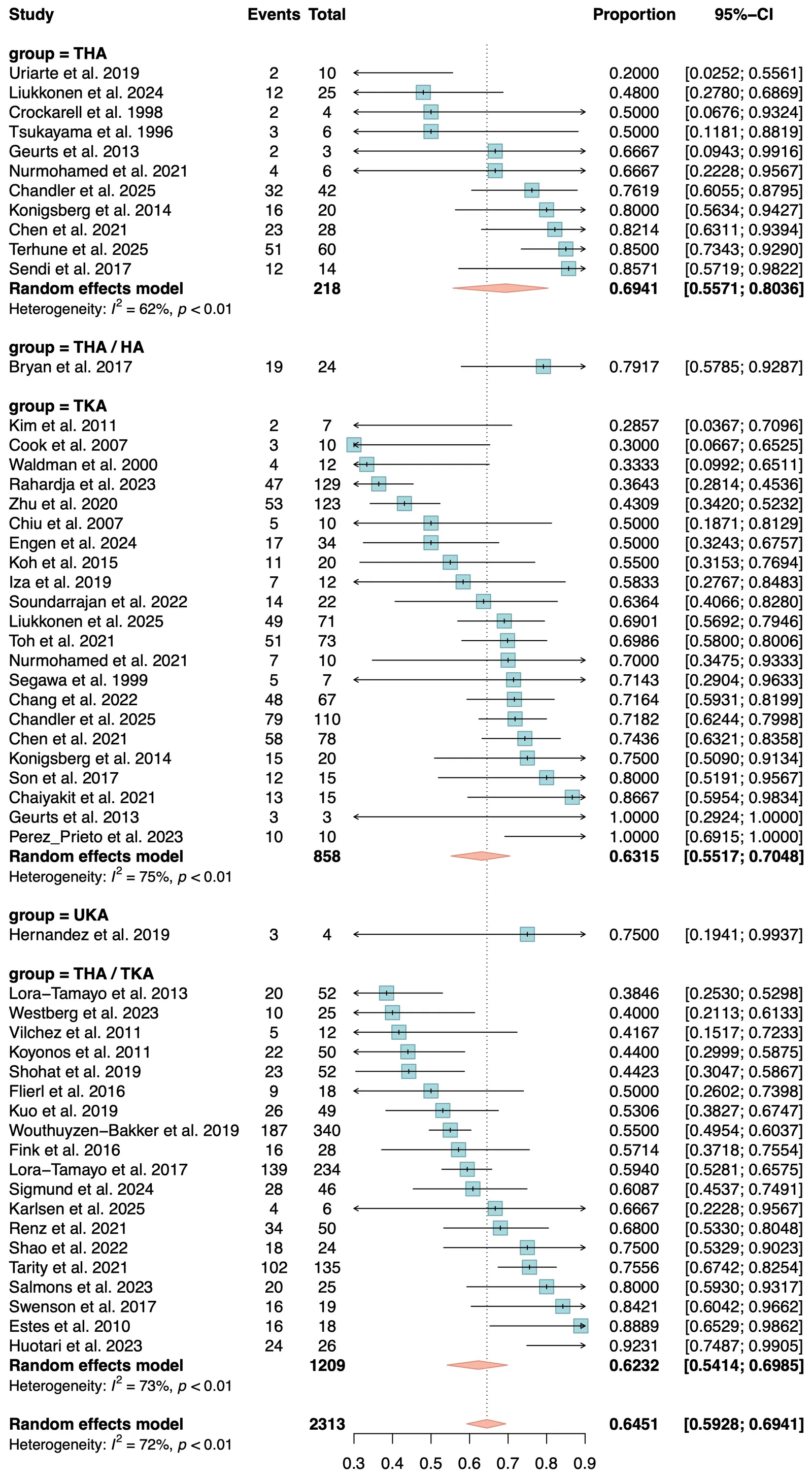
Forest plot showing the pooled success rate of DAIR for acute hematogenous PJI across all included studies, along with subgroup analyses for THA and TKA.

**Table 1:** Study characteristics.

Study	Country	Design	Hematogenous	Joint	Population,	Age,	Men,	Follow-up,
			infection		n	year	n	years
			interval					
			to index					
			procedure					
Soundarrajan et al. (2023)	India	Retrospective	>4 weeks	TKA	22	67.4±9.85	10 (45 %)	4.9±2.4
Chen et al. (2021)	USA	Prospective	>3 months	78 TKA; 28 THA	106	67.2±11.6	58 (55 %)	4.9 (3.5–7)
Huotari et al. (2023)	Finland	Retrospective	>3 months	15 TKA; 11 THA	26	71.2 (38–86)	12 (46 %)	4.5 (15d–9.3)
Rahardja et al. (2023)	Australia and New Zealand	Prospective	>3 months	TKA	129	70 (36–90)	75 (58 %)	2
Karlsen et al. (2025)	Norway	Prospective	>4 weeks	4 TKA; 2 THA	6	63±14	3 (50 %)	4.2
Liukkonen et al. (2024)	Finland	Retrospective	>3 months	THA	25	NA	NA	1
Tarity et al. (2021)	USA	Retrospective	>6 weeks	84 TKA; 51 THA	135	66.1±12.0	71 (53 %)	0.3
Geurts et al. (2013)	Netherlands	Retrospective	>4 weeks	3 TKA; 3 THA	6	NA	NA	2.8 (1m–23)
Westberg et al. (2023)	Norway	Retrospective	>4 weeks	TKA, THA	25	75 (43–92)	NA	1
Segawa et al. (1999)	USA	Retrospective	>4 weeks	TKA	7	NA	NA	3.3 (0.3–5.5)
Bryan et al. (2017)	USA	Retrospective	>4 weeks	THA, HA	24	NA	NA	6 (2–15)
Chiu and Chen (2007)	China	Prospective	>4 weeks	TKA	10	73.9	4 (40 %)	6.6 (3–12)
Cook et al. (2007)	Canada	Retrospective	NA	TKA	10	67	NA	10
Crockarell et al. (1998)	USA	Retrospective	>4 weeks	THA	4	59	2 (50 %)	6.3 (0.14–22)
Tsukayama et al. (1996)	USA	Retrospective	>4 weeks	THA	6	NA	NA	2.6 (0.5–4.0)
Estes et al. (2010)	USA	Retrospective	NA	TKA, THA	18	67 (28–91)	NA	3.5 (1.2–7.5)
Fink et al. (2017)	Germany	Retrospective	NA	20 TKA; 8 THA	28	NA	NA	3.5 (2–11)
Flierl et al. (2017)	USA	Retrospective	NA	TKA, THA	18	62 (32–88)	NA	1.1 (0.25–2.5)
Hernandez et al. (2019)	USA	Retrospective	NA	UKA	4	NA	NA	At least 2y
Uriarte et al. (2019)	Spain	Retrospective	NA	THA	10	NA	NA	4 (1–12)
Engen et al. (2024)	USA	Retrospective	>4 weeks	TKA	34	NA	NA	2
Kim et al. (2011)	Korea	Retrospective	>4 weeks	TKA	7	65±9.4	NA	At least 2 yr
Koh et al. (2015)	Korea	Retrospective	>4 weeks	TKA	20	67.6 (44–80)	NA	At least 2 yr
Konigsberg et al. (2014)	USA	Retrospective	>6 months	20 TKA; 20 THA	40	60 (25–86)	22 (55 %)	At least 2 yr
Koyonos et al. (2011)	USA	Retrospective	>4 weeks	34 TKA; 16 THA	50	63.5 (60.6–66.3)	28 (56 %)	4.5 (1–9.6)
Son et al. (2017)	Korea	Retrospective	>4 weeks	TKA	15	70.8 (35–86)	NA	2.5 (2–2.9)
Kuo et al. (2019)	USA	Retrospective	>3 months	42 TKA; 7 THA	49	NA	26 (53 %)	At least 1 yr
Lora-Tamayo et al. (2013)	Spain	Retrospective	NA	38 TKA; 13 THA	52	74 (65–79)	21 (40 %)	2
Salmons et al. (2023)	USA	Retrospective	>4 weeks	11 TKA; 14 THA	25	71 (41–90)	NA	7 (2–15)
Renz et al. (2022)	Germany	Retrospective	>4 weeks	TKA, THA	50	74 (36–92)	NA	1.7 (0.2m–7.5)
Lora-Tamayo et al. (2017)	Spain	Retrospective	>3 months	TKA, THA	242	72 (65–78)	111 (46 %)	2.2 (1.4–3.7)
Shao et al. (2022)	China	Retrospective	>3 months	TKA, THA	24	62.5 (54–75)	NA	3.2 (2–7.6)
Chaiyakit et al. (2021)	Thailand	Retrospective	>4 weeks	TKA	12	65.9 (48.0–82.0)	3 (25 %)	7.8 (4.7–11.1)
Iza et al. (2019)	Spain	Retrospective	>3 months	TKA	12	73 (57–83)	NA	3.4 (1–12)
Pérez-Prieto et al. (2023)	Spain	Prospective	>3 and <12 months	TKA	10	72.6	2 (20 %)	8.5±2.4
Chang et al. (2022)	Korea	Retrospective	>3 months	TKA	67	74±9.2	12 (18 %)	2
Toh et al. (2021)	Singapore	Retrospective	>3 months	TKA	73	64.6±9.74	NA	1.9 (0.5–7.8)
Sendi et al. (2017)	Switzerland	Retrospective	>3 months	THA	14	73.2 (44.6–87.7)	NA	4.0 (1.4–10)
Shohat et al. (2019)	USA	Retrospective	>3 months	43 TKA; 9 THA	52	68.7±11.6	27 (52 %)	1

**Table 1:** Continued.

Study	Country	Design	Hematogenous	Joint	Population,	Age,	Men,	Follow-up,
			infection		n	year	n	years
			interval					
			to index					
			procedure					
Swenson et al. (2018)	USA	Retrospective	>4 weeks	TKA, THA	19	NA	NA	2.89 (0.5–6.3)
Vilchez et al. (2011)	Spain	Retrospective	>3 months	10 TKA; 2 THA	12	74 (8.9)	6 (50 %)	1.4±1.2
Waldman et al. (2000)	USA	Prospective	>6 months	TKA	12	71.3	5 (42 %)	4.2
Wouthuyzen-Bakker et al. (2019a)	International multicenter	Retrospective	>3 months	247 TKA; 93 THA	340	NA	175 (51 %)	2.8 (1.3–4.6)
Zhu et al. (2021)	New Zealand	Retrospective	>4 weeks	TKA	102	NA	NA	6.9±3.69
			>3 months		123			
Nurmohamed et al. (2021)	Netherlands	Prospective	NA	10 TKA; 6 THA	16	NA	NA	At least 1 yr
Sigmund et al. (2024)	UK	Retrospective	>4 weeks	TKA, THA	46	NA	NA	At least 1 yr
Liukkonen et al. (2025)	Finland	Retrospective	>3 months	TKA	71	NA	NA	At least 1 yr
Chandler et al. (2025)	USA	Retrospective	>3 months	110 TKA; 42 THA	152	66.0±10.7	79 (53 %)	6.4 (3.9–8.5)
Terhune et al. (2025)	USA	Retrospective	>4 weeks	THA	60	66±13.7	NA	7 (2–15)

TKA: total knee arthroplasty; THA: total hip arthroplasty; HA: hemi-arthroplasty; UKA: unicompartmental knee arthroplasty; NA: not available; n : number.

**Table 2:** Summary of the Joanna Briggs Institute (JBI) critical appraisal of the included studies, by domain.

Domain	JBI checklist item	Yes,	No,
		n (%)	n (%)
Case definition and identification	1. Clear criteria for inclusion	41 (84)	8 (16)
	2. Condition measured in a standard, reliable way	41 (84)	8 (16)
	3. Valid methods used to identify the condition	41 (84)	8 (16)
Participant inclusion	4. Consecutive inclusion of participants	46 (94)	3 (6)
	5. Complete inclusion of participants	46 (94)	3 (6)
Reporting	6. Clear reporting of participant demographics	45 (92)	4 (8)
	7. Clear reporting of clinical information	8 (16)	41 (84)
	8. Clear reporting of outcomes or follow-up results	47 (96)	2 (4)
	9. Clear reporting of site or clinic demographics	45 (92)	4 (8)
Statistical analysis	10. Appropriate statistical analysis	49 (100)	0 (0)

Values are numbers of studies (% of 49). Study-level ratings are provided in Table S2.

### 3.6 Subgroup analyses

Subgroup analyses showed somewhat higher success rates in hips than in knees, a finding that is clinically informative given potential differences in implant anatomy, soft-tissue exposure, and treatment response between joint types (Fig. 2). Among patients with total hip arthroplasty (THA), the pooled success rate was 69.4 % (159 of 218; 95 % CI: 55.7 %–80.4 %), whereas among patients with total knee arthroplasty (TKA), success was achieved in 513 of 858 cases (pooled prevalence: 63.2 %; 95 % CI: 55.2 %–70.5 %; Fig. 2).

When studies were stratified by publication period, outcomes improved over time. Studies published before 2010 reported a pooled success rate of 44.9 % (95 % CI: 31.7 %–58.9 %; k=6 ), those published from 2010 to 2019 had a pooled success rate of 60.2 % (95 % CI: 51.7 %–68.0 %; k=21 ), and those published between 2020 and 2025 achieved the highest pooled success rate at 68.7 % (95 % CI: 61.4 %–75.2 %; k=22 ). This temporal trend was statistically significant ( P=0.0098 ), suggesting that contemporary DAIR management may be associated with improved outcomes (Fig. 3).

By contrast, subgroup analyses based on study definitions of acute hematogenous PJI should be interpreted more cautiously. Studies using a threshold of more than 4 weeks after the index procedure reported a pooled success rate of 64.1 % (334 of 553; 95 % CI: 55.6 %–71.8 %), and those using a threshold of more than 3 months reported a similar pooled success rate of 64.1 % (903 of 1519; 95 % CI: 55.6 %–69.6 %; Fig. S6). However, these findings remain exploratory because of heterogeneity in study definitions, overlap in clinical classification methods, and inconsistencies in reporting across studies.

Regional subgroup analysis showed pooled success rates of 66.3 % (95 % CI: 57.7 %–73.9 %) in North America, 62.8 % (95 % CI: 52.5 %–72.0 %) in Europe, and 60.9 % (95 % CI: 49.8 %–71.0 %) in the Asia–Pacific region. The international multicenter study had a success rate of 55.0 % (95 % CI: 49.5 %–60.4 %). There was no statistically significant difference in pooled success rates across geographic subgroups ( P=0.127 ; Fig. S7).

**Figure 3:**
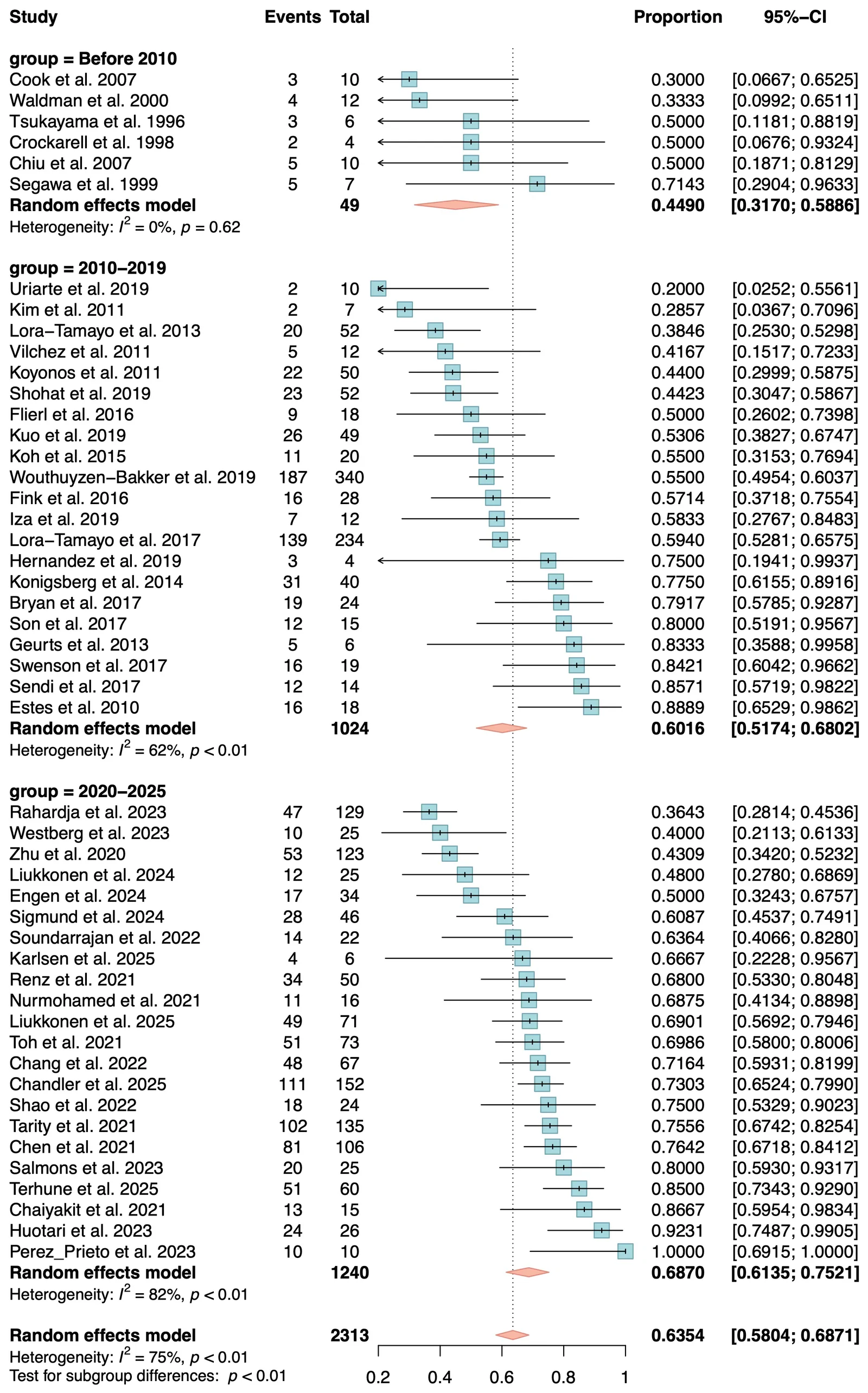
Forest plot illustrating the pooled success rates of DAIR in acute hematogenous PJI across publication periods (pre-2010, 2010–2019, and 2020–2025), demonstrating a significant temporal improvement in success rate.

### 3.7 Restrictive sensitivity analysis

To address the heterogeneity in diagnostic criteria and follow-up duration, a restrictive sensitivity analysis was performed including only studies that provided detailed diagnostic criteria for acute hematogenous PJI, included at least 10 patients, and reported at least 1 year of follow-up. This analysis comprised 31 studies with 1780 patients and yielded a pooled treatment success rate of 62.5 % (95 % CI: 56.4 %–68.2 %; $I^{2}=76.3$ %). These findings were broadly consistent with the primary analysis (Fig. S8).

### 3.8 Meta-regression analyses

In meta-regression analyses adjusted for joint type, a higher proportion of *S. aureus* infections among the studies was significantly associated with lower DAIR success rates ( β=-3.35 , 95 % CI: -5.19 to -1.52 ; P=0.0003 ; k=23 ), while mean duration of antibiotic therapy was significantly associated with higher success rates ( β=0.035 , 95 % CI: 0.014–0.055; P=0.0009 ; k=18 ). In contrast, symptom-to-DAIR interval ( β=-0.058 , 95 % CI: -0.200 to 0.084; P=0.424 ; k=18 ); modular component exchange rate ( β=0.148 , 95 % CI: -1.34 to 1.64; P=0.846 ; k=34 ); and joint age, defined as the interval from index arthroplasty to DAIR ( β=-0.068 , 95 % CI: -0.464 to 0.328; P=0.737 ; k=16 ) were not significantly associated with success rate (Fig. S9).

### 3.9 Reported risk factors for DAIR failure

Reported predictors of DAIR failure were grouped into infection-related, timing-related, and procedure-related factors to improve clinical interpretability (Table S4).

*Infection-related factors.* *S. aureus* infection was the most consistently reported infection-related predictor of failure and was identified as a significant risk factor in seven studies using multivariable regression analyses. Polymicrobial infection was also associated with failure in some studies, although the supporting evidence was less consistent.

*Timing-related factors.* Prolonged symptom duration before DAIR was one of the most frequently reported timing-related predictors of treatment failure. In addition, delayed diagnosis or longer time to intervention was associated with poorer outcomes in several studies, supporting the importance of prompt recognition and early surgical management.

*Procedure-related factors.* Exchange of modular components was associated with improved treatment success in three studies, suggesting a potential procedural benefit in selected patients. When reported, surgeon specialization also appeared to be relevant, with treatment by non-specialist surgeons being associated with higher failure rates in at least one study.

## 4 Discussion

This systematic review and meta-analysis found that DAIR controls infection in about two-thirds of patients with acute hematogenous PJI. Outcomes were better in more recent studies than in older reports, and *S. aureus* infection and delayed intervention were the most consistent predictors of failure. To our knowledge, this is the largest focused synthesis of this treatment strategy specifically for acute hematogenous periprosthetic joint infection.

Our findings are broadly consistent with previous literature. Abbaszadeh et al. (2026) reported a success rate of about 60.9 % for acute hematogenous cases close to our pooled estimate of 64.5 %, but that review included only eight studies. By including 49 studies and more recent data, the present review provides a broader and more clinically focused estimate for this distinct infection subtype.

These findings characterize the reported success of DAIR in selected patients with acute hematogenous PJI. However, because this meta-analysis was based on non-comparative studies, the observed success rate should not be interpreted as evidence of comparative effectiveness relative to one- or two-stage-revision strategies. Treatment failure still occurs in about one-third of cases, and so careful candidate selection remains essential. Patients with *S. aureus* infection or delayed symptom-to-surgery intervals should be approached with particular caution. The higher success in hip arthroplasty than in knee arthroplasty and the significant temporal improvement over time are clinically informative, but the central message remains the same: early recognition, timely surgery, and multidisciplinary management are critical.

Our subgroup analysis showed a higher success rate for THA than for TKA (69.4 % vs. 63.2 %), consistent with prior literature. A 2021 meta-regression analysis reported pooled DAIR success rates of 70 % for hip and 63 % for knee PJI (Gerritsen et al., 2021), supporting the view that DAIR may achieve modestly better outcomes in hips than in knees.

DAIR outcomes improved significantly over time, with pooled success rates increasing from 44.9 % before 2010 to 60.2 % in 2010–2019 and 68.7 % in 2020–2025 ( P=0.0098 ). However, this temporal association should not be interpreted as evidence of improved therapeutic effectiveness over time. Differences in diagnostic criteria, patient selection, microbiological characteristics, timing of intervention, surgical practices, and antibiotic management may have contributed to the observed trend. Era-stratified analyses of *S. aureus* prevalence, mean symptom-to-DAIR interval, and modular component exchange were not feasible because these variables were inconsistently reported across studies. Furthermore, the available data did not permit a reliable multivariable meta-regression simultaneously accounting for publication period, *S. aureus* prevalence, symptom-to-DAIR interval, and modular component exchange. Therefore, the observed temporal trend should be considered to be hypothesis-generating rather than evidence of a causal improvement in DAIR outcomes.

This review reported several predictors of DAIR failure, most notably *S. aureus* infection and prolonged symptom duration, while modular component exchange was associated with higher success. *S. aureus* accounted for 34.2 % of infections and was consistently linked to failure, with seven multivariate studies confirming it as an independent risk factor. Reported odds ratios ranged from 3.6 to 4.7 (Zhu et al., 2021; Toh et al., 2021). Our meta-regression analysis also demonstrated a study-level association between a higher proportion of *S. aureus* infections and lower success rates. Prolonged symptom duration was also a recurrent predictor of failure, with five studies demonstrating its significance; Uriarte et al. identified an 11 d threshold beyond which outcomes worsened (Uriarte et al., 2019). These findings are biologically plausible as delayed intervention encounters mature biofilms with increased antimicrobial tolerance.

Three studies in our review found that modular component exchange was associated with improved outcomes, although the broader literature remains mixed. A large Swedish registry study of 575 patients reported higher success with component exchange than without exchange (71.4 % vs. 55.5 %; HR 0.51; P<0.001 ) (Svensson et al., 2020). In contrast, a meta-regression analysis of 65 studies (Gerritsen et al., 2021), consistent with our study-level meta-regression, found no significant association between the rate of component exchange and overall DAIR success. Deng et al. further reported that, among patients with sinus tracts, modular exchange was associated with significantly higher success rates (75.8 % vs. 47.4 %; P=0.038 ), suggesting that its value may be context dependent (Deng et al., 2021). Although most surgeons favored modular exchange when feasible, inconsistent reporting precluded formal meta-analysis. While biologically plausible due to improved debridement and biofilm removal, current evidence is insufficient for a definitive conclusion, and any benefit likely depends on clinical and operative factors.

Multivariate analyses identified several additional prognostic factors, including higher failure rates when DAIR was performed by non-specialist surgeons compared with hip specialists (85 % vs. 33 %) (Uriarte et al., 2019). Positive blood cultures (Kuo et al., 2019; Lora-Tamayo et al., 2017) and elevated preoperative inflammatory markers (e.g., ESR > 107.5 mm h^−1^ (Toh et al., 2021) or CRP > 65 mg L^−1^ (Zhu et al., 2021)) were also associated with poorer outcomes. Although the CRIME80 score has been proposed to guide DAIR selection in acute hematogenous PJI, it lacks external validation (Wouthuyzen-Bakker et al., 2019b). In our study-level meta-regression, a longer mean duration of antibiotic therapy was significantly associated with higher DAIR success rates, suggesting that the duration of antimicrobial treatment may be an additional factor associated with treatment outcomes. However, this finding should be interpreted cautiously because the analysis was conducted at the study level and may reflect differences in treatment protocols, patient selection, or other unmeasured study-level characteristics. Overall, optimal patient selection remains unclear and likely depends on a combination of patient, microbiologic, implant, soft-tissue, and treatment-related factors.

This review has several limitations. Most included studies were retrospective, with inherent risks of bias and confounding. Definitions of acute hematogenous PJI and treatment success varied substantially, limiting comparability. Classification as acute hematogenous PJI was based on the designation provided by the authors of each included study. As a documented well-functioning interval before symptom onset and explicit clinical or microbiological evidence of hematogenous seeding were not consistently required, some cohorts may have included acute infections of post-surgical rather than hematogenous origin. Accordingly, the pooled estimate should be interpreted as representing acute PJIs designated as hematogenous in the source literature, while the restrictive sensitivity analysis provides an estimate less susceptible to potential misclassification. A microorganism-specific analysis of treatment success was not feasible because few studies reported treatment outcomes stratified by causative organism specifically within the acute hematogenous PJI population. Key variables, including symptom duration, microbiology, modular exchange, bacteremia, and antibiotic regimens, were inconsistently reported, precluding detailed subgroup analyses and robust prognostic modeling. Furthermore, the timing of DAIR in relation to bacteremia and treatment or source control of the remote infection was insufficiently reported, preventing assessment of whether DAIR was performed during ongoing bacteremia or after control of the remote infectious focus and whether the duration of treatment of the remote source influenced outcomes. In particular, the source of hematogenous seeding was reported in only a limited number of studies and was generally not available at the individual-patient level; therefore, its potential influence on treatment outcomes could not be reliably evaluated. Accordingly, the pooled success rate should be interpreted as an average across heterogeneous practices rather than as a patient-specific prediction.

Future research should prioritize prospective comparative studies to clarify the role of DAIR relative to one- and two-stage revision in acute hematogenous PJI; the development of validated risk prediction models to improve patient selection; and the broader adoption of standardized definitions, outcomes, and reporting frameworks to strengthen evidence synthesis. Future studies should also systematically report the clinical and microbiological evidence supporting hematogenous seeding, including the documented remote source of infection, to facilitate more precise characterization of patient populations and evaluation of source-specific treatment outcomes.

## 5 Conclusion

DAIR achieved infection control in approximately two-thirds of reported cases of acute hematogenous PJI. Because this synthesis was non-comparative, these data describe the success rate of DAIR and not its effectiveness relative to one- or two-stage revision. For clinical practice, the findings argue for prompt recognition and early surgical intervention, with particular caution in patients with *S. aureus* infection or delayed presentation, in whom reported success rates were consistently lower. Longer antibiotic therapy was associated with higher success rates in study-level meta-regression, although this finding should not be interpreted as causal. Overall, patient selection, prompt intervention, and individualized multidisciplinary management should guide the decision to pursue DAIR rather than relying on the pooled success rate as a patient-specific prediction.

## Supplement

10.5194/jbji-11-673-2026-supplement The supplement related to this article is available online at https://doi.org/10.5194/jbji-11-673-2026-supplement.

## Data Availability

The extracted study-level dataset generated during this review is available from the corresponding author upon reasonable request. It is not publicly posted because the database was manually compiled from published reports and requires study-level verification and harmonization before reuse.
