# Supplementary material for: Success of debridement, antibiotics, and implant retention for acute hematogenous periprosthetic joint infection: a systematic review and meta-analysis
Source: J Bone Jt Infect. 2026 Nov 9;11(6):673–86. doi: 10.5194/jbji-11-673-2026 (PMC13747197; doi:10.5194/jbji-11-673-2026)
Supplement: Supplementary file 1 [file jbji-11-673-2026-supplement.pdf]

Supplement of J. Bone Joint Infect., 11, 673–686, 2026
https://doi.org/10.5194/jbji-11-673-2026-supplement
© Author(s) 2026. CC BY 4.0 License.

Supplement of

Success of debridement, antibiotics, and implant retention for acute hematogenous periprosthetic joint infection: a systematic review and meta-analysis

Sina Esmaeili et al.

Correspondence to: Seyed Mohammad Javad Mortazavi (smjmort@yahoo.com)

The copyright of individual parts of the supplement might differ from the article licence.

Table S1. Complete search strategy

Database	Search string
**PubMed**	(PJI[Title/Abstract] OR "Periprosthetic joint infection*"[Title/Abstract] OR "Peri-prosthetic joint infection*"[Title/Abstract] OR "Prosthesis related Infection*"[Title/Abstract] OR "Prosthesis-related Infection*"[Title/Abstract] OR "Prosthetic joint infection*"[Title/Abstract] OR "Prosthesis-associated infection*"[Title/Abstract] OR "Prosthesis associated infection*"[Title/Abstract] OR "Periprosthetic infection*"[Title/Abstract] OR "Peri-prosthetic infection*"[Title/Abstract] OR "Prosthetic infection*"[Title/Abstract] OR "Prosthesis infection*"[Title/Abstract] OR "Prosthesis-Related Infections"[Mesh]) AND (Hematogenous[Title/Abstract] OR Haematogenous[Title/Abstract] OR Blood-borne[Title/Abstract] OR "Blood borne"[Title/Abstract] OR "Hematogenetic"[Title/Abstract] OR "Blood-Borne Pathogens"[Mesh])
**Scopus**	TITLE-ABS(PJI) OR TITLE-ABS("Periprosthetic joint infection*") OR TITLE-ABS("Peri-prosthetic joint infection*") OR TITLE-ABS("Prosthesis related Infection*") OR TITLE-ABS("Prosthesis-related Infection*") OR TITLE-ABS("Prosthetic joint infection*") OR TITLE-ABS("Prosthesis-associated infection*") OR TITLE-ABS("Prosthesis associated infection*") OR TITLE-ABS("Periprosthetic infection*") OR TITLE-ABS("Peri-prosthetic infection*") OR TITLE-ABS("Prosthetic infection*") OR TITLE-ABS("Prosthesis infection*")) AND (TITLE-ABS(Hematogenous) OR TITLE-ABS(Haematogenous) OR TITLE-ABS("Blood-borne") OR TITLE-ABS("Blood borne") OR TITLE-ABS(Hematogenetic))
**Web of Science**	(TS=PJI OR TS="Periprosthetic joint infection*" OR TS="Peri-prosthetic joint infection*" OR TS="Prosthesis related Infection*" OR TS="Prosthesis-related Infection*" OR TS="Prosthetic joint infection*" OR TS="Prosthesis-associated infection*" OR TS="Prosthesis associated infection*" OR TS="Periprosthetic infection*" OR TS="Peri-prosthetic infection*" OR TS="Prosthetic infection*" OR TS="Prosthesis infection*") AND (TS=Hematogenous OR TS=Haematogenous OR TS="Blood-borne" OR TS="Blood borne" OR TS=Hematogenetic)

**Embase**	'PJI':ti,ab,kw OR 'Periprosthetic joint infection*':ti,ab,kw OR 'Peri-prosthetic joint infection*':ti,ab,kw OR 'Prosthesis related infection*':ti,ab,kw OR 'Prosthesis-related infection*':ti,ab,kw OR 'Prosthetic joint infection*':ti,ab,kw OR 'Prosthesis-associated infection*':ti,ab,kw OR 'Prosthesis associated infection*':ti,ab,kw OR 'Periprosthetic infection*':ti,ab,kw OR 'Peri-prosthetic infection*':ti,ab,kw OR 'Prosthetic infection*':ti,ab,kw OR 'Prosthesis infection*':ti,ab,kw<br>'periprosthetic joint infection'/exp<br>#1 OR #2<br>'Hematogenous':ti,ab,kw OR 'Haematogenous':ti,ab,kw OR 'Blood-borne':ti,ab,kw OR 'Blood borne':ti,ab,kw OR 'Hematogenetic':ti,ab,kw<br>'blood-borne infection'/exp<br>#4 OR #5<br>#3 AND #6

Table S2. Quality assessment using the Joanna Briggs Institute (JBI) critical appraisal tool

Study	Q1	Q2	Q3	Q4	Q5	Q6	Q7	Q8	Q9	Q10
Soundarrajan et al. 2022	Yes	Yes	Yes	Yes	Yes	Yes	No	Yes	Yes	Yes
Chen et al. 2021	Yes	Yes	Yes	Yes	Yes	Yes	No	Yes	Yes	Yes
Huotari et al. 2023	Yes	Yes	Yes	Yes	Yes	Yes	No	Yes	Yes	Yes
Rahardja et al. 2023	Yes	Yes	Yes	Yes	Yes	Yes	No	Yes	Yes	Yes
Karlsen et al. 2025	Yes	Yes	Yes	Yes	Yes	No	No	Yes	No	Yes
Liukkonen et al. 2024	Yes	Yes	Yes	Yes	Yes	Yes	No	Yes	Yes	Yes
Tarity et al. 2021	Yes	Yes	Yes	Yes	Yes	Yes	No	Yes	Yes	Yes
Geurts et al. 2013	Yes	Yes	Yes	Yes	Yes	Yes	No	No	Yes	Yes
Westberg et al. 2023	Yes	Yes	Yes	Yes	Yes	Yes	No	Yes	Yes	Yes
Segawa et al. 1999	Yes	Yes	Yes	Yes	Yes	Yes	No	Yes	Yes	Yes
Bryan et al. 2017	Yes	Yes	Yes	Yes	Yes	Yes	No	Yes	Yes	Yes
Chiu et al. 2007	Yes	Yes	Yes	Yes	Yes	Yes	Yes	Yes	Yes	Yes
Cook et al. 2007	No	No	No	Yes	Yes	Yes	Yes	Yes	Yes	Yes
Crockarell et al. 1998	Yes	Yes	Yes	Yes	Yes	Yes	No	Yes	Yes	Yes
Tsukayama et al. 1996	Yes	Yes	Yes	Yes	Yes	Yes	No	Yes	Yes	Yes
Estes et al. 2010	No	No	No	Yes	Yes	Yes	No	Yes	Yes	Yes
Fink et al. 2016	No	No	No	Yes	Yes	Yes	Yes	Yes	Yes	Yes
Flierl et al. 2016	No	No	No	Yes	Yes	Yes	No	Yes	Yes	Yes
Hernandez et al. 2019	No	No	No	Yes	Yes	Yes	No	Yes	Yes	Yes
Uriarte et al. 2019	No	No	No	Yes	Yes	Yes	No	Yes	Yes	Yes
Engen et al. 2024	Yes	Yes	Yes	No	No	Yes	No	Yes	Yes	Yes
Kim et al. 2011	Yes	Yes	Yes	Yes	Yes	Yes	Yes	Yes	Yes	Yes
Koh et al. 2015	Yes	Yes	Yes	Yes	Yes	Yes	No	Yes	Yes	Yes
Konigsberg et al. 2014	Yes	Yes	Yes	Yes	Yes	Yes	No	Yes	Yes	Yes
Koyonos et al. 2011	Yes	Yes	Yes	Yes	Yes	Yes	No	Yes	Yes	Yes
Son et al. 2017	Yes	Yes	Yes	Yes	Yes	No	No	Yes	No	Yes
Kuo et al. 2019	Yes	Yes	Yes	Yes	Yes	Yes	No	Yes	Yes	Yes
Lora-Tamayo et al. 2013	No	No	No	Yes	Yes	Yes	Yes	Yes	Yes	Yes
Salmons et al. 2023	Yes	Yes	Yes	Yes	Yes	Yes	No	No	Yes	Yes
Renz et al. 2021	Yes	Yes	Yes	Yes	Yes	Yes	Yes	Yes	Yes	Yes
Lora-Tamayo et al. 2017	Yes	Yes	Yes	Yes	Yes	No	No	Yes	No	Yes
Shao et al. 2022	Yes	Yes	Yes	No	No	Yes	No	Yes	Yes	Yes
Chaiyakit et al. 2021	Yes	Yes	Yes	Yes	Yes	Yes	No	Yes	Yes	Yes
Iza et al. 2019	Yes	Yes	Yes	Yes	Yes	Yes	No	Yes	Yes	Yes
Pérez-Prieto et al. 2023	Yes	Yes	Yes	Yes	Yes	Yes	No	Yes	Yes	Yes
Chang et al. 2022	Yes	Yes	Yes	Yes	Yes	Yes	No	Yes	Yes	Yes
Toh et al. 2021	Yes	Yes	Yes	Yes	Yes	Yes	No	Yes	Yes	Yes
Sendi et al. 2017	Yes	Yes	Yes	Yes	Yes	Yes	No	Yes	Yes	Yes
Shohat et al. 2019	Yes	Yes	Yes	Yes	Yes	Yes	No	Yes	Yes	Yes
Swenson et al. 2017	Yes	Yes	Yes	Yes	Yes	Yes	Yes	Yes	Yes	Yes
Vilchez et al. 2011	Yes	Yes	Yes	Yes	Yes	Yes	No	Yes	Yes	Yes
Waldman et al. 2000	Yes	Yes	Yes	No	No	Yes	No	Yes	Yes	Yes
Wouthuyzen-Bakker et al. 2019	Yes	Yes	Yes	Yes	Yes	Yes	Yes	Yes	Yes	Yes

Zhu et al. 2020	Yes	Yes	Yes	Yes	Yes	Yes	No	Yes	Yes	Yes
Nurmohamed et al. 2021	No	No	No	Yes	Yes	Yes	No	Yes	Yes	Yes
Sigmund et al. 2024	Yes	Yes	Yes	Yes	Yes	Yes	No	Yes	Yes	Yes
Liukkonen et al. 2025	Yes	Yes	Yes	Yes	Yes	Yes	No	Yes	Yes	Yes
Chandler et al. 2025	Yes	Yes	Yes	Yes	Yes	No	No	Yes	No	Yes
Terhune et al. 2025	Yes	Yes	Yes	Yes	Yes	Yes	No	Yes	Yes	Yes

Q1. Were there clear criteria for inclusion in the case series?
Q2. Was the condition measured in a standard, reliable way for all participants included in the case series?
Q3. Were valid methods used for identification of the condition for all participants included in the case series?
Q4. Did the case series have consecutive inclusion of participants?
Q5. Did the case series have complete inclusion of participants?
Q6. Was there clear reporting of the demographics of the participants in the study?
Q7. Was there clear reporting of clinical information of the participants?
Q8. Were the outcomes or follow up results of cases clearly reported?
Q9. Was there clear reporting of the presenting site(s)/clinic(s) demographic information?
Q10. Was statistical analysis appropriate?

Table S3. Microorganisms, Timing, Antibiotic Therapy, and Modular Component Exchange Rate

Study	Infective organisms reported, n(%)	Time intervals from onset of symptoms (days) to DAIR	Time interval from index procedure and DAIR (joint age, year)	Exchange modular components, %	Duration of IV antibiotic therapy, weeks	Duration of Oral antibiotic therapy, weeks
Soundarrajan et al. 2022	NA	NA	1-6 months (4)<br>7 months-1 year (4)<br>1 year-3 years (7)<br>3years-5 years (5)<br>More than 5 years (2)	NA	2	6
Chen et al. 2021	*S. aureus* 26 (24.5%)<br>b-hemolytic *Streptococcus* 9 (8.5%)<br>Coagulase NG *Staphylococcus* 7 (6.6%)<br>Polymicrobial 9 (8.5%)<br>Culture negative 24 (22.6%)	NA	NA	NA	6	NA
Huotari et al. 2023	*S. aureus* 26 (100%)	6.8 (3-18)	NA	100%	4.5 ± 1	10.9 (8–39.6)
Rahardja et al. 2023	*S. aureus* 63 (49%)<br>Gram negative 17 (13%)<br>Polymicrobial 22 (17%)<br>Culture negative 12 (9%)	NA	NA	NA	NA	NA
Karlsen et al. 2025	*S. aureus* 3 (50%)<br>*Streptococcus* spp 2 (33%)<br>*Enterococcus faecalis* 1 (17%)	NA	NA	100%	2	4
Liukkonen et al. 2024	*S. aureus* 25 (100%)	NA	NA	100%	NA	NA
Geurts et al. 2013	NA	7.5 d	NA	0%	NA	NA
Westberg et al. 2023	*S.aureus* 16 (37%)<br>*Streptococcus* spp 13 (30%)<br>CoNS 3 (7%)<br>Polymicrobial 0	7 (2-120)	6.5 (0.3–23)	72%	2	4
Segawa et al. 1999	NA	NA	NA	100%	6	NA
Bryan et al. 2017	NA	6 (1-21)	2.4 (36w-14)	70%	6	NA
Chiu et al. 2007	Coagulase NG *Staphylococcus* 4 (40%)<br>*S. aureus* 4 (40%)<br>Group B *strep* 1 (10%)<br>*Candida* 1 (10%)	NA	NA	NA	At least 6	NA
Cook et al. 2007	*S. aureus* 3 (30%)<br>Group B *strep* 1 (10%)<br>*Pseudomonas* 2 (20%)<br>*Streptococcus pneumoniae* 2 (20%)<br>Coagulase NG *Staphylococcus* 1 (10%)<br>Culture negative 1 (10%)	NA	2.8 (0.8-6)	90%	4.1 (2d-10.3)	10 (0.9-54)
Tsukayama et al. 1996	Gram-positive *cocci*	2-7	NA	100%	6	NA
Estes et al. 2010	NA	NA	NA	100%	6	36
Fink et al. 2016	*S. aureus* 7 (25%)<br>*MSSE* 4 (14.3%)<br>*E. coli* 2 (7.1%)<br>*Enterococcus faecalis* 2 (7.1%)	NA	NA	100%	2	4

	Streptococcus agalactiae 2 (7.1%)<br>*Staphylococcus lugdunensis* 2 (7.1%)					
Flierl et al. 2016	NA	NA	NA	100%	At least 6	variable course
Hernandez et al. 2019	*S. aureus* 2 (50%)<br>*Staphylococcus lugdunensis* 1 (25%)<br>Culture negative 1 (25%)	NA	0.7	NA	6	NA
Uriarte et al. 2019	NA	NA	2 (1.2m-17)	NA	2.5 w (0.5-8)	5.5 (1-8)
Engen et al. 2024	NA	NA	NA	100%	6	12-, 24-, or 48-weeks
Kim et al. 2011	Coagulase-positive *staphylococci* 2 (28%)<br>Coagulase-negative *staphylococci* 2 (28%)<br>Culture negative 2 (28%)<br>*Enterobacter* 1 (14%)	NA	NA	100%	6	NA
Koh et al. 2015	NA	8 (4-26)	1.4 (0.4-12)	94%	6	NA
Konigsberg et al. 2014	*S. aureus* 15 (38%)<br>*Streptococcal species* 15 (38%)<br>*E. coli* 2 (5%)<br>Polymicrobial 2 (5%)<br>Culture negative 2 (5%)	5.4 (1-28)	4.7 (0.5-15.5)	100%	6	42 (6-104)
Koyonos et al. 2011	*Staphylococcus* organism 20 (40%)<br>*S. aureus* 17 (34%)<br>Methicillin-resistant 6 (12%)<br>Gram-negative organism 6 (12%)<br>*Staphylococcus epidermis* 3 (6%)<br>Culture negative 8 (16%)	NA	NA	NA	6	NA
Son et al. 2017	NA	3.3 (1-5)	NA	100%	At least 4	2
Kuo et al. 2019	*Staphylococcal* species 26 (53%)<br>*S. aureus* 24 (48%)<br>*Streptococcus* 15 (30%)<br>Gram negative 3 (6%)<br>*Staphylococcus epidermidis* 2 (4%)<br>Polymicrobial 5 (10%)	NA	NA	100%	6	NA
Lora-Tamayo et al. 2013	*S. aureus* 52 (100%)<br>Polymicrobial 1 (2%)	NA	NA	63%	NA	NA
Salmons et al. 2023	NA	NA	1.3 (4.4w-12)	NA	5 (3-6)	NA
Lora-Tamayo et al. 2017	*Streptococci* 242 (100%)<br>Polymicrobial 11 (5%)	5 (2-12)	NA	55%	3 ± 2.9	13 (8.3-24.4)
Shao et al. 2022	NA	NA	NA	most cases	least 2	At least 4
Chaiyakit et al. 2021	*S. aureus* 2 (17%)<br>Group A *streptococcus* 2 (17%)<br>Group B *streptococcus* 1 (8%)<br>Culture negative 10 (83%)	4.5 (1-10)	2.5 (0.2–5.8)	100%	6	12
Iza et al. 2019	NA	15 (3-21)	2.2 (0.3-8)	100%	12 (4–53)	
Pérez-Prieto et al. 2023	*S. aureus* 3 (30%)<br>*E. coli* 3 (30%)<br>Coagulase-negative *staphylococci* 2 (20%)<br>Culture negative 2 (20%)	2.75 ± 1.8	4.7 ± 3.6	100%	11.4 ± 1.9	
Chang et al. 2022	*S. aureus* 15 (22%)	8.3 ± 8.2	1.3 (0.7–3.6)	100%	4-6	NA

	Streptococcus 15 (22%)<br>Gram negative 9 (13%)<br>Polymicrobial 3 (4%)<br>Culture negative 14 (21%)					
Sendi et al. 2017	*S. aureus* 5 (36%)<br>*Streptococcus* spp. 8 (57%)	NA	NA	59%	1-2	10-11
Shohat et al. 2019	*S. aureus* 26 (50%)<br>*Streptococcus* 17 (33%)<br>*VRE, MRSA,* or *MRSE* 7 (13%)<br>Gram-negative 5 (10%)<br>Polymicrobial 7 (13%)	NA	NA	100%	6	NA
Swenson et al. 2017	NA	NA	NA	100%	At least 6	NA
Vilchez et al. 2011	*S. aureus* 12 (100%)<br>Poly 0 (0%)	4.7 ± 3.7	4 ± 3.2	100%	1 ± 8.3	16.5 ± 7.9
Waldman et al. 2000	*S. aureus* 7 (58%)<br>*S. epidermidis* 4 (33%)<br>*b-hemolytic streptococcus* 1 (8%)	4.4 (1-7)	3.3 (0.5-7.8)	NA	6	NA
Wouthuyzen-Bakker et al. 2019	*S. aureus* 141 (41%)<br>*Streptococcus* species 97 (29%)<br>Gram negative 50 (15%)<br>*Enterococcus* species 11 (3%)	NA	NA	NA	12	
Zhu et al. 2020	NA	NA	NA	81%	At least 6	NA
Nurmohamed et al. 2021	NA	12 (0-83)	NA	NA	12	
Sigmund et al. 2024	*S. aureus* 18 (39.1%)<br>*Streptococcus* species 14 (30.4%)<br>Polymicrobial 3 (6.5%)<br>Culture negative 4 (8.7%)	NA	NA	NA	NA	
Chandler et al. 2025	*S. aureus* 25 (16.4%)<br>*Streptococcus* species 22 (14.5%)<br>Polymicrobial 46 (30.3%)<br>Culture negative 25 (16.4%)	NA	NA	100%	6	12-24
Terhune et al. 2025	*S. aureus* 14 (23.3%)<br>*Streptococcus* species 14 (23.3%)<br>Polymicrobial 4 (6.7%)<br>Culture negative 10 (16.7%)	NA	1.1 (5w-21)	60%	6 (4-9)	NA

DAIR, Debridement, Antibiotics, and Implant Retention; NA, Not Available; n, Number

Table S4. Treatment Outcome and Risk Factors for Failure

Study	Successful Patients, n (%)	Success Definition	Failed Patients, n (%)	Failure Definition	Surgical option for failed patients	Failure risk factors
Soundarrajan et al. 2022	14/22 (64%)	Absence of laboratory signs (ESR, CRP, WBC, Synovial fluid analysis)	8/22 (36%)	(1) Need any further surgery (2) Need antibiotic suppression therapy (3) Death related to infection	Two-stage revision<br>Re-debridement<br>Arthrodesis	Univariate analysis:<br>Symptom duration
Chen et al. 2021	81/106 (76%)	NA	25/106 (24%)	(1) Need any further surgery (2) Infection recurrence caused by the same organism strain (3) Death related to infection	Single-stage revision (5)<br>Two-stage revision (7)<br>Arthrodesis (12)<br>Amputation (1)	Multivariate analysis:<br>Diabetes mellitus<br>Polymicrobial infections
Huotari et al. 2023	24/26 (92%)	(1) Retaining the original prosthesis (2) No death due to infection (3) No active signs of infection	2/26 (8%)	NA	Two-stage revision	NA
Rahardja et al. 2023	47/129 (36%)	(1) Retaining the original prosthesis (2) No death due to infection (3) No active signs of infection (4) no ongoing use of antibiotics	82/129 (64%)	NA	NA	Univariate analysis:<br>Time to PJI Diagnosis
Karlsen et al. 2025	4/6 (67%)	(1) Absence of laboratory (ESR, CRP, WBC) and clinical signs (2) No signs of implant loosening	2/6 (33%)	(1) Need any further surgery (2) Need antibiotic suppression therapy	NA	Univariate analysis:<br>PJI following revision (vs. primary)
Liukkonen et al. 2024	12/25 (48%)	NA	13/25 (52%)	(1) Need any further surgery (due to any reason)	NA	Univariate analysis:<br>DAIR (vs. revision)
Tarity et al. 2021	102/135 (76%)	NA	33/135 (34%)	(1) Need revision surgery	NA	Multivariate analysis:<br>*Staphylococcus* infection
Geurts et al. 2013	5/6 (83%)	(1) Absence of laboratory (ESR, CRP, WBC), clinical, and radiological signs	1/6 (17%)	NA	Revision	NA
Westberg et al. 2023	10/25 (40%)	(1) Absence of laboratory and clinical signs (2) No signs of implant loosening	15/25 (60%)	(1) Need any further surgery (2) Infection recurrence caused by the same organism strain (3) Death related to infection	Two-stage revision (6)<br>Suppressive antibiotic (3)<br>Amputation (2)<br>DAIR and resection arthroplasty (2)<br>New DAIR (1)<br>Girdlestone procedure (1)	NA
Segawa et al. 1999	5/7 (71%)	(1) Absence of clinical signs with a functional knee with a total joint prosthesis in place	2/7 (29%)	NA	Suppressive antibiotic (1)<br>Arthrodesis (1)	NA
Bryan et al. 2017	19/24 (79%)	(1) Absence of laboratory signs (ESR, CRP)	5/24 (21%)	(1) Need revision surgery (2) Infection recurrence caused by the same organism strain (3) Death related to infection	NA	NA
Chiu et al. 2007	5/10 (50%)	(1) Absence of clinical signs (2) Preservation of prosthetic function (3) Retaining the original prosthesis	5/10 (50%)	(1) Need revision surgery (2) Infection recurrence	Two-stage revision (3)<br>Fusion (2)	NA

Cook et al. 2007	3/10 (30%)	NA	7/10 (70%)	NA	Resection arthroplasty and reimplantation (4)<br>Resection arthroplasty and reimplantation plus I & D with liner exchange (3)<br>Amputation (1)	NA
Crockarell et al. 1998	2/4 (50%)	NA	2/4 (50%)	(1) Need revision surgery	NA	NA
Tsukayama et al. 1996	3/6 (50%)	(1) Absence of clinical signs	3/6 (50%)	NA	Revision (2)<br>Resection (1)	NA
Estes et al. 2010	16/18 (89%)	(1) Absence of laboratory (ESR, CRP) and clinical signs	2/18 (11%)	(1) Need revision surgery (2) Infection recurrence (3) Need long-term antibiotic therapy	Long-term oral antibiotics (2)	NA
Fink et al. 2016	16/28 (57%)	(1) Absence of laboratory (CRP) and clinical signs	12/28 (43%)	(1) Infection recurrence	NA	Univariate analysis:<br>Time to PJI Diagnosis<br>Comorbidities score
Flierl et al. 2016	9/18 (50%)	NA	9/18 (50%)	NA	Two-stage revision<br>Suppressive antibiotic	NA
Hernandez et al. 2019	3/4 (75%)	(1) No need for reoperation	1/4 (25%)	(1) Infection recurrence	Two-stage revision (1)	NA
Uriarte et al. 2019	2/10 (20%)	(1) Absence of laboratory (ESR, CRP), clinical, and radiological signs (2) Prosthesis retention	8/10 (80%)	(1) Need revision surgery (2) Need long-term antibiotic therapy (3) Death related to infection	One-stage revision<br>Two-stage revision<br>Suppressive antibiotic	Univariate analysis:<br>Male sex<br>General orthopedic surgeon (vs. hip surgeon)<br>Symptom duration (11 days was a significant cut-off point for success)
Engen et al. 2024	17/34 (50%)	(1) Absence of laboratory signs (WBC, ESR, CRP)	17/34 (50%)	(1) Need any further surgery	NA	NA
Kim et al. 2011	2/7 (29%)	NA	5/7 (71%)	NA	Revision (4)<br>Two times revision arthroplasty and arthrodesis (1)	NA
Koh et al. 2015	11/20 (55%)	(1) Prosthesis retention (2) no ongoing use of antibiotics	9/20 (45%)	(1) Need antibiotic suppression therapy (2) Need revision surgery, arthrodesis, resection arthroplasty, or amputation	Two-stage revision (6)<br>Suppressive antibiotic (1)<br>Fusion or amputation (2)	Univariate analysis:<br>Acute hematogenous (vs. early post-surgical)<br>Symptom duration
Konigsberg et al. 2014	33/42 (79%)	NA	9/42 (21%)	(1) Need any further surgery (2) evidence of persistent infection (e.g. a sinus tract), or prosthetic loosening	Two-stage revision (8)<br>Resection (1)	NA
Koyonos et al. 2011	22/50 (44%)	(1) Absence of laboratory signs (ESR, CRP)	28/50 (56%)	(1) Need any further surgery (2) Need long-term antibiotic therapy	Further surgery (22)<br>Long-term oral antibiotics (2)	Multivariate analysis:<br>*Staphylococcal* infection
Son et al. 2017	12/15 (80%)	(1) Absence of laboratory and radiological signs	3/15 (20%)	(1) Need any further surgery (2) Need long-term antibiotic therapy (3) Death related to infection	Two-stage revision (3)	NA
Kuo et al. 2019	26/49 (53%)	(1) Absence of laboratory signs (ESR, CRP)	23/49 (47%)	(1) Need any further surgery (2) Infection recurrence caused by the same organism strain (3) Death related to infection	NA	Multivariate analysis:<br>Positive blood culture

Lora-Tamayo et al. 2013	20/52 (38%)	NA	32/52 (62%)	(1) Need revision surgery (2) Death related to infection (3) Need for extra debridements (4) Need long-term antibiotic therapy	NA	Multivariate analysis:<br>*Staphylococcal* infection<br>Symptom duration
Salmons et al. 2023	20/25 (80%)	NA	5/25 (20%)	(1) Need revision surgery	NA	NA
Renz et al. 2021	34/50 (68%)	NA	16/50 (32%)	(1) Death related to infection (2) Infection recurrence	NA	Univariate analysis:<br>DAIR (vs. implant removal)
Lora-Tamayo et al. 2017	139/234 (59%)	NA	95/234 (41%)	(1) Need revision surgery (2) Death related to infection (3) Need for extra debridements (4) Need long-term antibiotic therapy	NA	Multivariate analysis:<br>Rheumatoid arthritis<br>Bacteremia<br>---<br>Exchange of removable components was associated with a favorable outcome
Shao et al. 2022	18/24 (75%)	NA	6/24 (25%)	(1) Fistula, drainage, or pain, and infection recurrence caused by the same organism (2) Need any further surgery (3) Death related to infection	NA	Multivariate analysis:<br>Symptom duration
Chaiyakit et al. 2021	13/15 (87%)	(1) Absence of laboratory (ESR, CRP), clinical, and radiological signs (2) Synovial fluid analysis	2/15 (13%)	(1) Nonhealed wound with or without sinus tract, wound drainage, and persistent pain (2) Need any further surgery (3) Death related to infection (4) Need long-term antibiotic therapy	Two-stage revision	NA
Iza et al. 2019	7/12 (58%)	(1) Absence of laboratory (ESR, CRP) and clinical signs (2) Prosthesis retention (3) no ongoing use of antibiotics	5/12 (42%)	(1) Need any further surgery (2) Infection recurrence caused by the same organism strain (3) Death related to infection	Two-stage revision<br>Suppressive antibiotic	Multivariate analysis:<br>*Staphylococcus* aureus
Pérez-Prieto et al. 2023	10/10 (100%)	NA	0/10 (0%)	(1) Need any further surgery (2) Infection recurrence (3) Death related to infection (4) Suppressive antibiotic	NA	NA
Chang et al. 2022	48/67 (72%)	NA	19/67 (28%)	(1) Repeated DAIR or two-stage revision	Repeated DAIR<br>Two-stage revision	NA
Toh et al. 2021	51/73 (70%)	Chronic antibiotic suppression post-DAIR were considered a success	22/73 (30%)	(1) Need revision surgery, arthrodesis, or amputation	Revision<br>Arthrodesis<br>Amputation	Multivariate analysis:<br>*Staphylococcal* infection<br>Higher preoperative ESR (above 107.5)
Sendi et al. 2017	12/14 (86%)	NA	2/14 (14%)	NA	Suppressive antibiotic (1)<br>Single-stage revision	NA
Shohat et al. 2019	23/52 (44%)	NA	29/52 (56%)	(1) Need any further surgery (2) Infection recurrence caused by the same organism strain (3) Infection recurrence	NA	Multivariate analysis:<br>Higher preoperative ESR<br>Comorbidities score
Swenson et al. 2017	16/19 (84%)	NA	3/19 (16%)	NA	NA	NA
Vilchez et al. 2011	5/12 (42%)	(1) Absence of clinical signs (2) Prosthesis retention	7/12 (58%)	(1) Infection recurrence caused by the same organism strain (2) aseptic loosening	NA	Multivariate analysis:<br>Hematogenous infections (vs. early post-surgical)

Waldman et al. 2000	4/12 (33%)	NA	8/12 (66%)	NA	Fusion<br>Two-stage revision	NA
Wouthuyzen-Bakker et al. 2019	187/340 (55%)	NA	153/340 (45%)	(1) Need revision surgery (2) Death related to infection (3) Need for suppressive antibiotic	Revision (55)<br>Suppressive antibiotic (24)	Multivariate analysis:<br>*Staphylococcus aureus*<br>DAIR (vs. implant removal)<br>---<br>Exchange of removable components was associated with a favorable outcome
Zhu et al. 2020	41/102 (40%)<br>53/123 (43%)	NA	61/102 (60%)<br>70/123 (57%)	(1) Need revision surgery (2) Death related to infection (3) Need for suppressive antibiotic	NA	Multivariate analysis:<br>Higher preoperative CRP (optimum threshold value of 65 mg/L)<br>*Staphylococcus aureus*<br>Gram-negative infections<br>---<br>Exchange of removable components and the lack of intraoperative purulence were associated with a favorable outcome
Nurmohamed et al. 2021	11/16 (69%)	(1) Absence of laboratory and clinical signs (2) no ongoing use of antibiotics	5/16 (31%)	(1) Need any further surgery (2) Need for suppressive antibiotic (3) Infection recurrence	NA	NA
Sigmund et al. 2024	28/46 (61%)	NA	18/46 (39%)	(1) Revision due to infection (2) Identification of microorganism in the affected joint (3) Need for suppressive antimicrobial therapy (4) Chronic sinus tract	NA	NA
Liukkonen et al. 2025	49/71 (69%)	(1) No need for reoperation	22/71 (31%)	(1) Reoperation due to any reason	NA	NA
Chandler et al. 2025	111/152 (73%)	(1) Absence of laboratory and clinical signs	42/152 (7%)	(1) Recurrent infection	NA	Multivariate analysis:<br>McPherson Systemic Host Grade C
Terhune et al. 2025	51/60 (85%)	NA	9/60 (15%)	(1) Infection recurrence	Two-stage revision<br>Irrigation and debridement<br>Girdlestone<br>Chronic suppression	Multivariate analysis:<br>McPherson Systemic Host Grade C

ESR, Erythrocyte Sedimentation Rate; CRP, C-Reactive Protein; WBC, White Blood Cell count; PJI, Periprosthetic Joint Infection; DAIR, Debridement, Antibiotics, and Implant Retention; NA, Not Available; n, Number

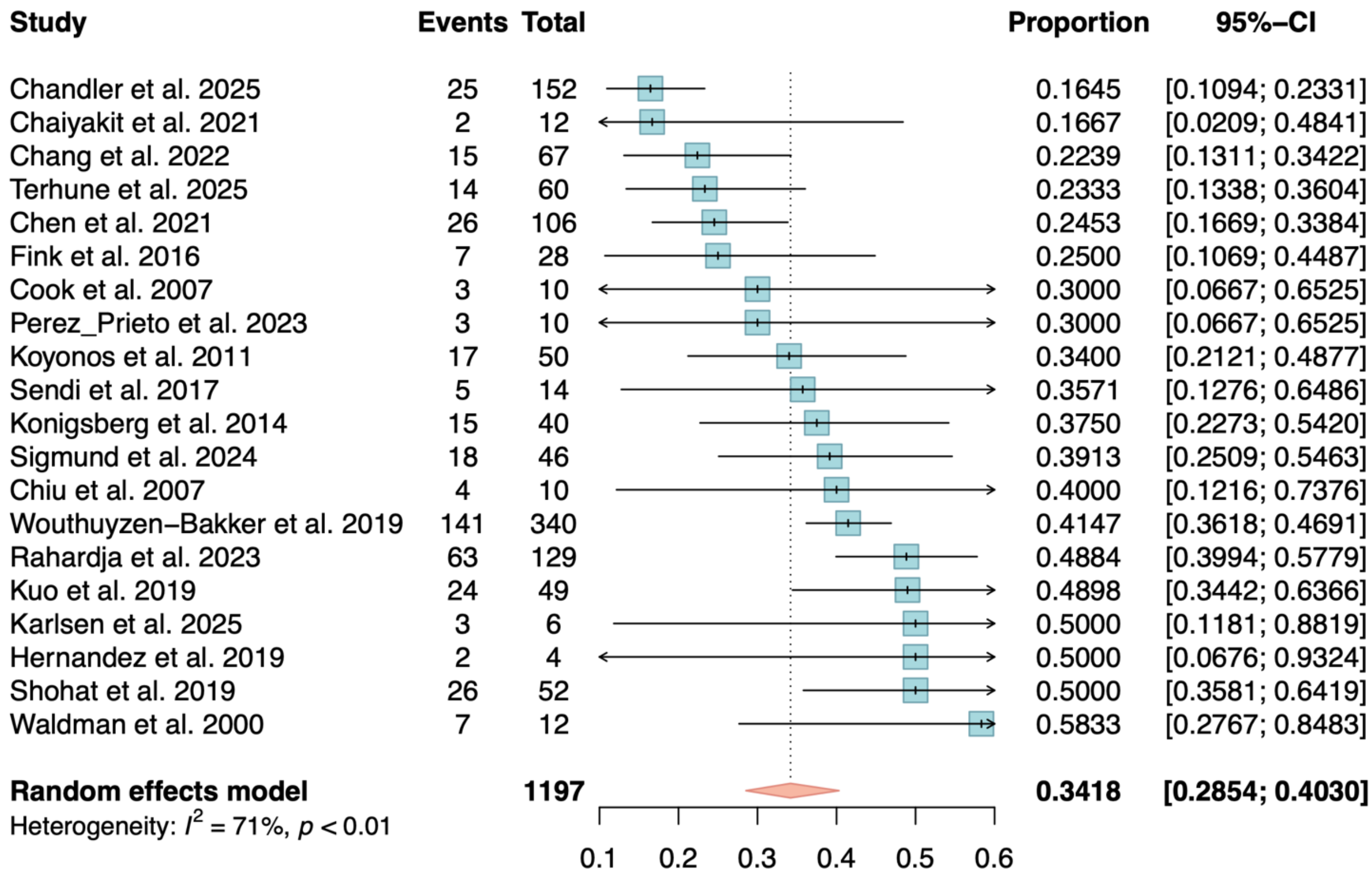

Figure S1. Forest plot illustrating the pooled proportion of Staphylococcus aureus as the causative organism in acute hematogenous PJI across included studies.

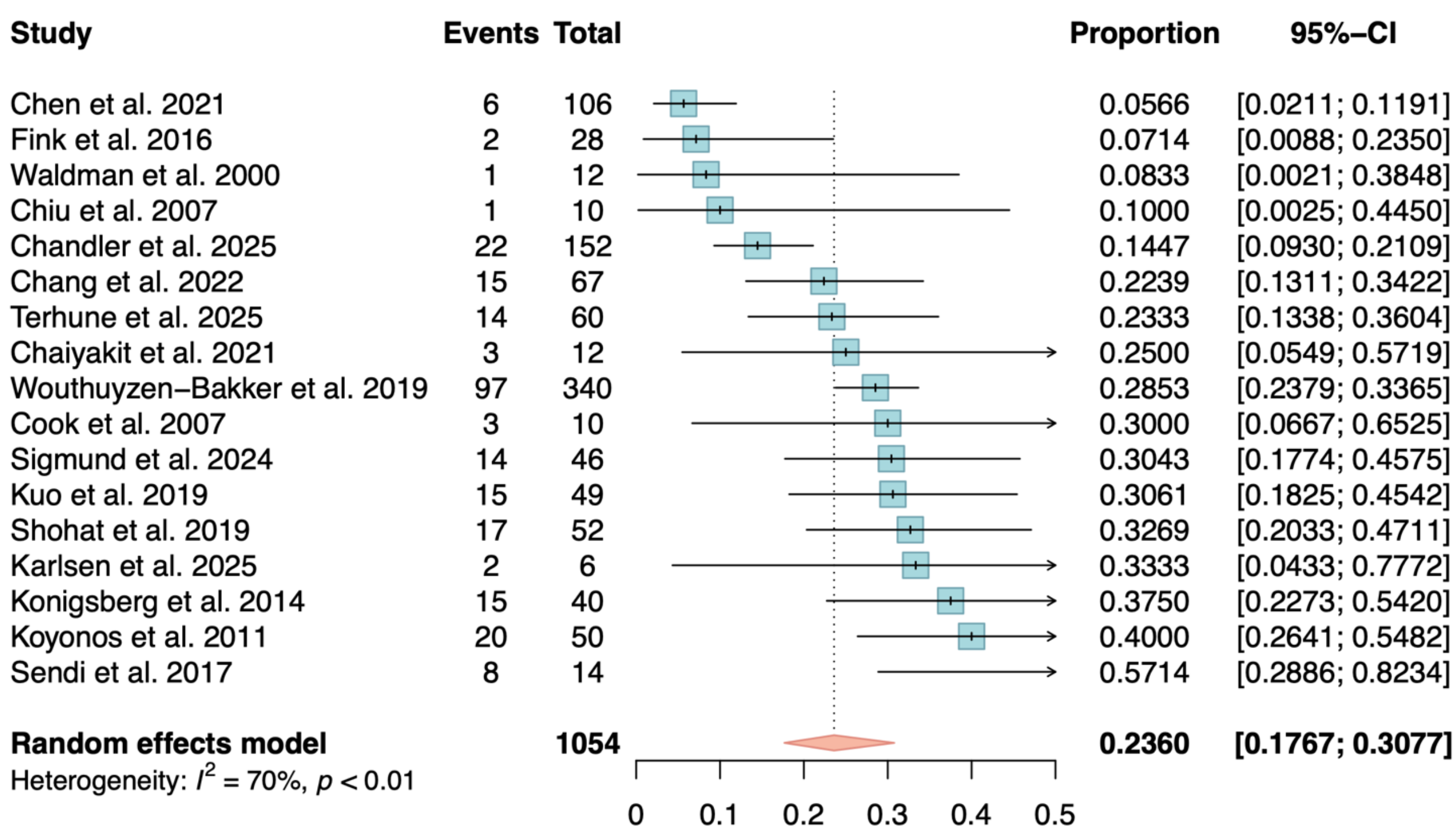

Figure S2. Forest plot illustrating the pooled proportion of Streptococcal species as the causative organism in acute hematogenous PJI across included studies.

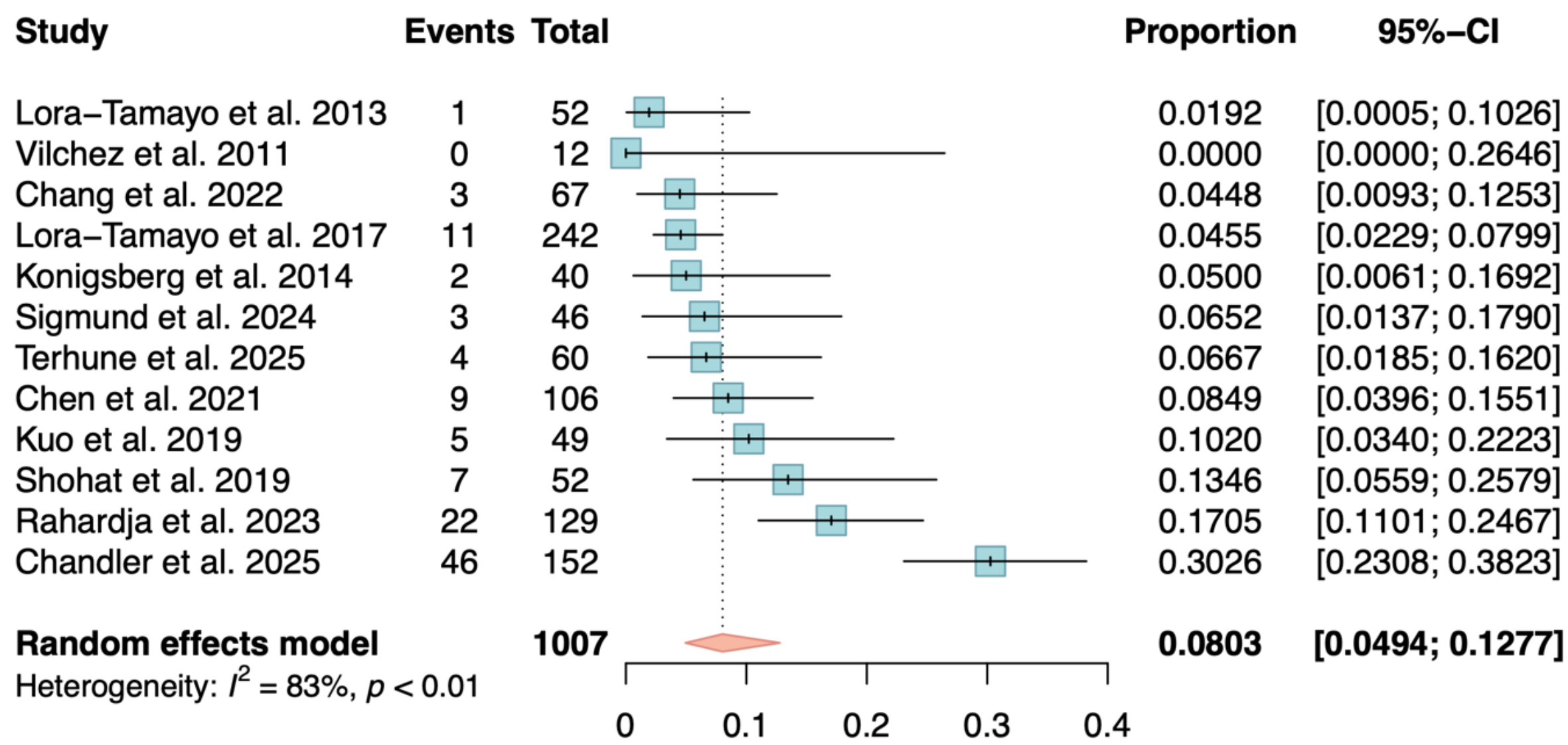

Figure S3. Forest plot illustrating the pooled proportion of polymicrobial infections among cases of acute hematogenous PJI across the included studies.

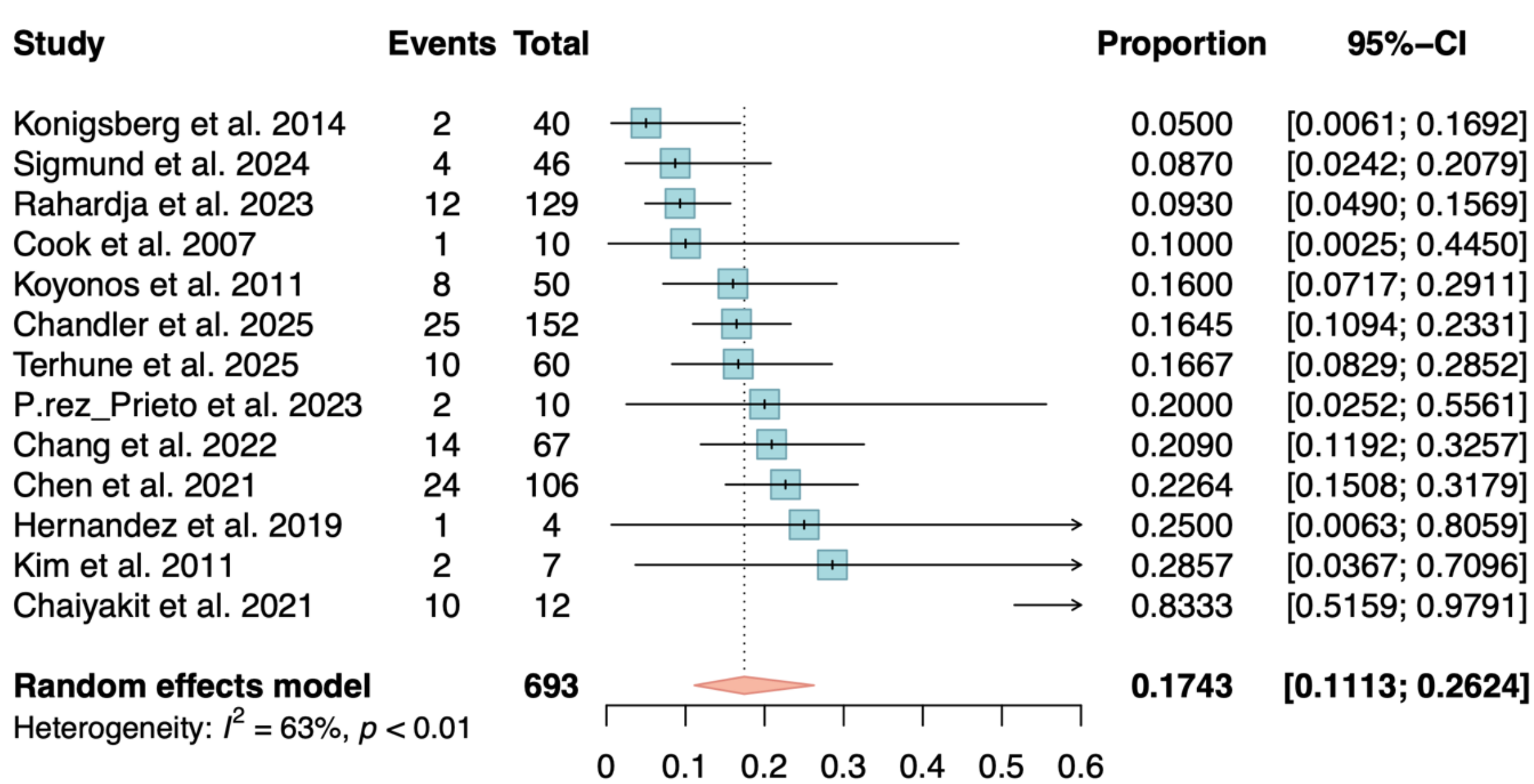

Figure S4. Forest plot illustrating the pooled proportion of culture-negative infections among cases of acute hematogenous PJI across the included studies.

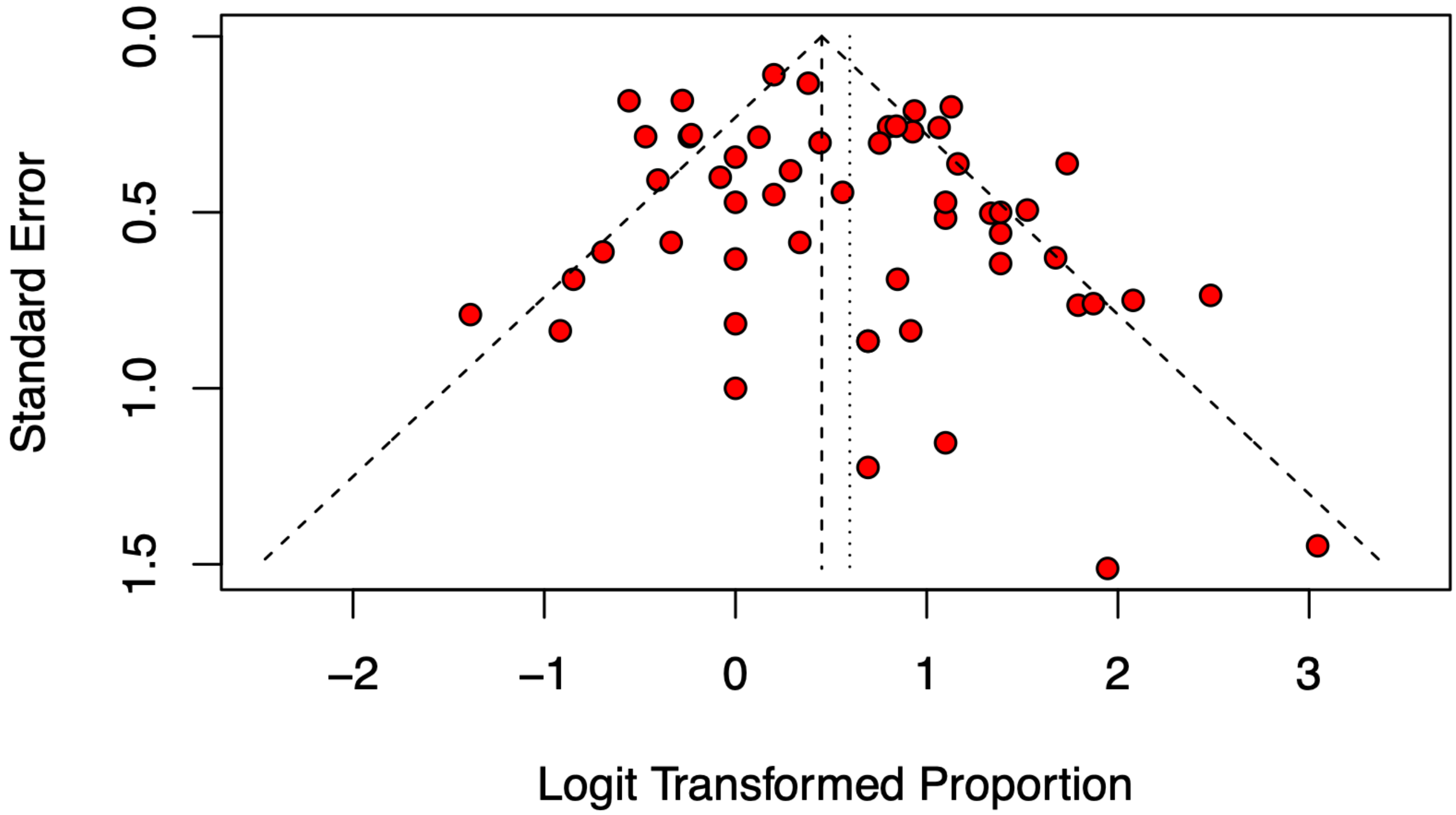

Figure S5. Funnel plot illustrating the assessment of publication bias using Egger's regression test

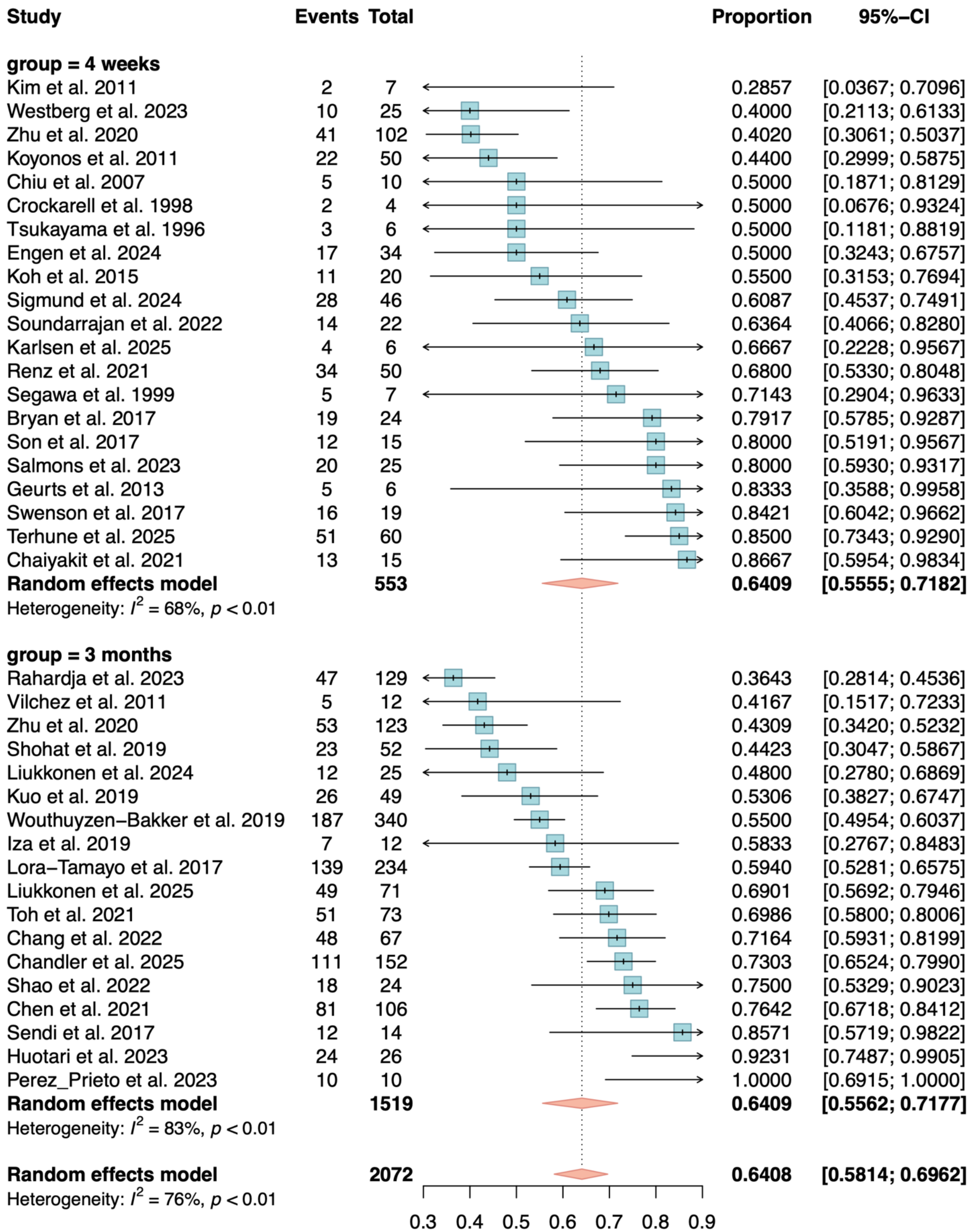

Figure S6. Forest plot showing the pooled success rates of DAIR in acute hematogenous PJI stratified by the definition of acute infection (four-week and three-month thresholds).

Study	Events	Total	Proportion	95%-CI
**North America**				
Cook et al. 2007	3	10	0.3000	[0.0667; 0.6525]
Waldman et al. 2000	4	12	0.3333	[0.0992; 0.6511]
Koyonos et al. 2011	22	50	0.4400	[0.2999; 0.5875]
Shohat et al. 2019	23	52	0.4423	[0.3047; 0.5867]
Crockarell et al. 1998	2	4	0.5000	[0.0676; 0.9324]
Engen et al. 2024	17	34	0.5000	[0.3243; 0.6757]
Flierl et al. 2016	9	18	0.5000	[0.2602; 0.7398]
Tsukayama et al. 1996	3	6	0.5000	[0.1181; 0.8819]
Kuo et al. 2019	26	49	0.5306	[0.3827; 0.6747]
Segawa et al. 1999	5	7	0.7143	[0.2904; 0.9633]
Chandler et al. 2025	111	152	0.7303	[0.6524; 0.7990]
Hernandez et al. 2019	3	4	0.7500	[0.1941; 0.9937]
Tarity et al. 2021	102	135	0.7556	[0.6742; 0.8254]
Chen et al. 2021	81	106	0.7642	[0.6718; 0.8412]
Konigsberg et al. 2014	31	40	0.7750	[0.6155; 0.8916]
Bryan et al. 2017	19	24	0.7917	[0.5785; 0.9287]
Salmons et al. 2023	20	25	0.8000	[0.5930; 0.9317]
Swenson et al. 2017	16	19	0.8421	[0.6042; 0.9662]
Terhune et al. 2025	51	60	0.8500	[0.7343; 0.9290]
Estes et al. 2010	16	18	0.8889	[0.6529; 0.9862]
**Random effects model**		**825**	**0.6630**	**[0.5770; 0.7394]**
Heterogeneity: $I^2 = 74.7\%$, $p < 0.0001$				
**Asia–Pacific**				
Kim et al. 2011	2	7	0.2857	[0.0367; 0.7096]
Rahardja et al. 2023	47	129	0.3643	[0.2814; 0.4536]
Zhu et al. 2020	53	123	0.4309	[0.3420; 0.5232]
Chiu et al. 2007	5	10	0.5000	[0.1871; 0.8129]
Koh et al. 2015	11	20	0.5500	[0.3153; 0.7694]
Soundarrajan et al. 2022	14	22	0.6364	[0.4066; 0.8280]
Toh et al. 2021	51	73	0.6986	[0.5800; 0.8006]
Chang et al. 2022	48	67	0.7164	[0.5931; 0.8199]
Shao et al. 2022	18	24	0.7500	[0.5329; 0.9023]
Son et al. 2017	12	15	0.8000	[0.5191; 0.9567]
Chaiyakit et al. 2021	13	15	0.8667	[0.5954; 0.9834]
**Random effects model**		**505**	**0.6094**	**[0.4984; 0.7101]**
Heterogeneity: $I^2 = 80.5\%$, $p < 0.0001$				
**Europe**				
Uriarte et al. 2019	2	10	0.2000	[0.0252; 0.5561]
Lora–Tamayo et al. 2013	20	52	0.3846	[0.2530; 0.5298]
Westberg et al. 2023	10	25	0.4000	[0.2113; 0.6133]
Vilchez et al. 2011	5	12	0.4167	[0.1517; 0.7233]
Liukkonen et al. 2024	12	25	0.4800	[0.2780; 0.6869]
Fink et al. 2016	16	28	0.5714	[0.3718; 0.7554]
Iza et al. 2019	7	12	0.5833	[0.2767; 0.8483]
Lora–Tamayo et al. 2017	139	234	0.5940	[0.5281; 0.6575]
Sigmund et al. 2024	28	46	0.6087	[0.4537; 0.7491]
Karlsen et al. 2025	4	6	0.6667	[0.2228; 0.9567]
Renz et al. 2021	34	50	0.6800	[0.5330; 0.8048]
Nurmohamed et al. 2021	11	16	0.6875	[0.4134; 0.8898]
Liukkonen et al. 2025	49	71	0.6901	[0.5692; 0.7946]
Geurts et al. 2013	5	6	0.8333	[0.3588; 0.9958]
Sendi et al. 2017	12	14	0.8571	[0.5719; 0.9822]
Huotari et al. 2023	24	26	0.9231	[0.7487; 0.9905]
Perez_Prieto et al. 2023	10	10	1.0000	[0.6915; 1.0000]
**Random effects model**		**643**	**0.6276**	**[0.5249; 0.7200]**
Heterogeneity: $I^2 = 58.1\%$, $p = 0.0014$				
**International–multicenter**				
Wouthuyzen–Bakker et al. 2019	187	340	0.5500	[0.4954; 0.6037]
**Random effects model**		**2313**	**0.6354**	**[0.5804; 0.6871]**
Heterogeneity: $I^2 = 75.1\%$, $p < 0.0001$				
Test for subgroup differences: $p = 0.1267$				

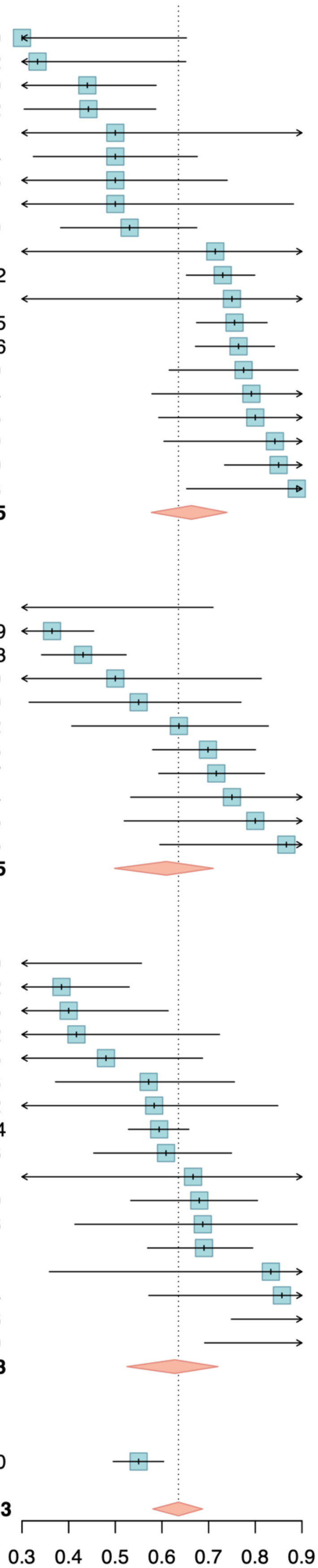

Figure S7. Forest plot of DAIR success rates by geographic region.

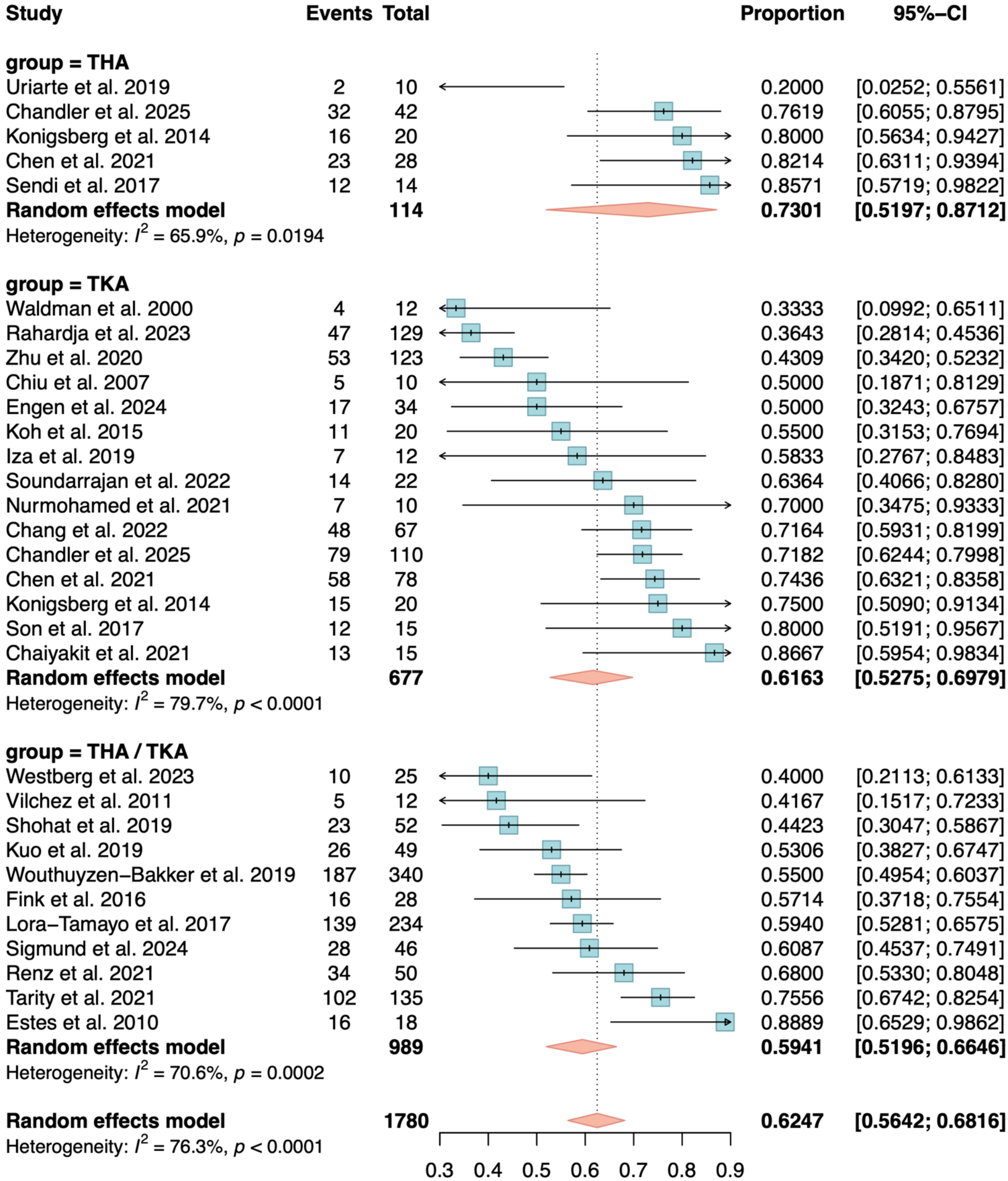

Figure S8. Forest plot of treatment success following DAIR for acute hematogenous PJI in the restrictive sensitivity analysis. Studies were limited to those with detailed diagnostic criteria, ≥10 patients, and ≥1 year of follow-up.

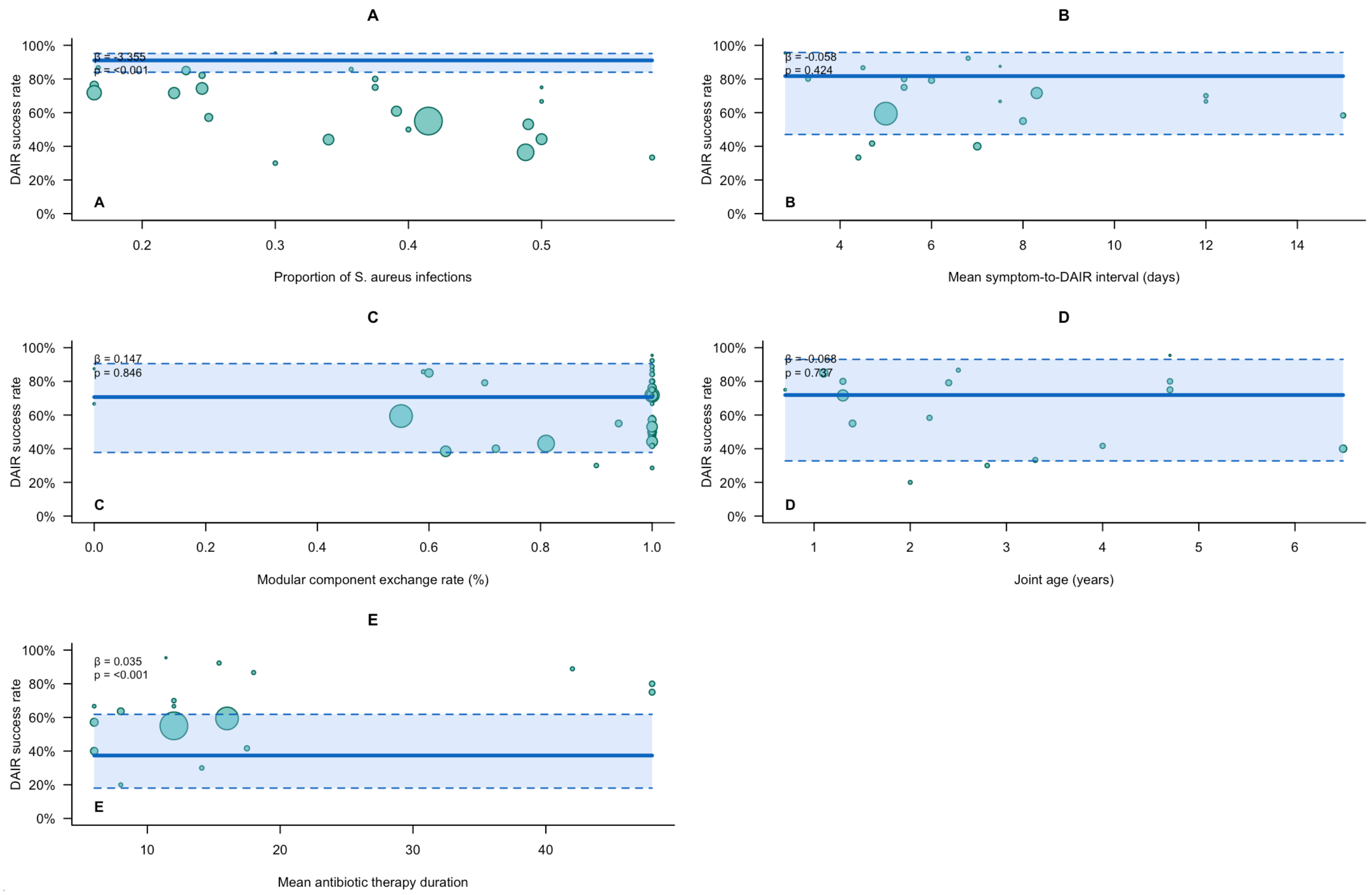

Figure S9. Meta-regression analyses of study-level DAIR success rates according to study-level clinical characteristics. Bubble plots illustrate the associations between DAIR success rate and (A) proportion of Staphylococcus aureus infections, (B) mean symptom-to-D
